# Lead-free hybrid perovskites for photovoltaics

**DOI:** 10.3762/bjnano.9.207

**Published:** 2018-08-21

**Authors:** Oleksandr Stroyuk

**Affiliations:** 1 Physikalische Chemie, Technische Universität Dresden, 01062 Dresden, Germany and L.V. Pysarzhevsky Institute of Physical Chemistry, National Academy of Sciences of Ukraine

**Keywords:** light harvesting, low-toxic materials, organo-inorganic perovskites, solar cells

## Abstract

This review covers the state-of-the-art in organo–inorganic lead-free hybrid perovskites (HPs) and applications of these exciting materials as light harvesters in photovoltaic systems. Special emphasis is placed on the influence of the spatial organization of HP materials both on the micro- and nanometer scale on the performance and stability of perovskite-based solar light converters. This review also discusses HP materials produced by isovalent lead(II) substitution with Sn^2+^ and other metal(II) ions, perovskite materials formed on the basis of M^3+^ cations (Sb^3+^, Bi^3+^) as well as on combinations of M^+^/M^3+^ ions aliovalent to 2Pb^2+^ (Ag^+^/Bi^3+^, Ag^+^/Sb^3+^, etc.). The survey is concluded with an outlook highlighting the most promising strategies for future progress of photovoltaic systems based on lead-free perovskite compounds.

## Review

### Introduction

The field of photovoltaics and photochemical light harvesting using nanocrystalline semiconductor materials is a thriving field of research that intersects physics, physical and material chemistry, photonics and photochemistry. The investment in photovoltaic solar cells has increased among other sustainable sources of electricity, whereby the market is dominated by silicon solar cells with top light-to-current conversion efficiencies reaching ≈27% [1]. As an alternative to the Si-based cells requiring a relatively thick absorber layer due to the indirect character of electron transitions in Si, direct-bandgap metal chalcogenide semiconductors have been employed as nanometer-thin-film light harvesters, such as Cu(Ga)InS(Se)_2_ or CdTe, showing a light conversion efficiency of up to 21% [1–2]. Progress in dye-sensitized solar cells (reaching ≈12% efficiency [1–2]) has stimulated attempts in using metal chalcogenide nanocrystals (NCs) as sensitizers in liquid-junction solar cells [3–4]. These systems have shown remarkable progress, improving from 0.1% a decade ago to over 12% in 2018 [5].

**Figure 1:**
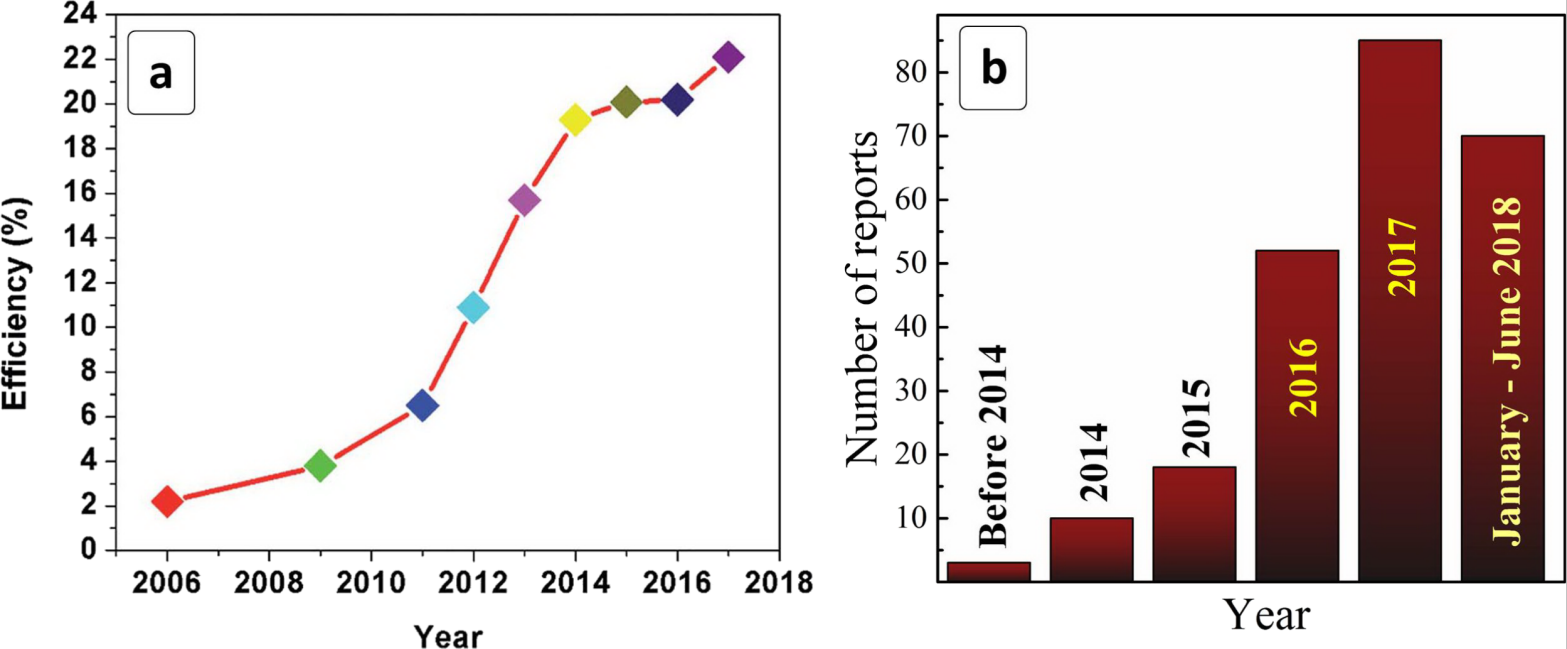
Temporal evolution of (a) the power conversion efficiency of lead-HP-based solar cells and (b) the number of publications on lead-free HPs. (a) Reprinted with permission from [27], copyright 2018 The Royal Society of Chemistry.

Simultaneously, a new rising star in semiconductor photovoltaics – hybrid organo–inorganic lead-based perovskites (MPbX_3_, where M = methylammonium (MA), formamidinium (FA), Cs; X = Cl, Br, I) – first employed only several years ago are making a fast progress increasing from ≈2% in 2006 to more than 20% starting from 2015 (Figure 1a) [6–31].

The avalanche progress of the hybrid perovskite (HP) photovoltaic system was documented in detail by numerous review papers covering all aspects of the preparative chemistry and photophysics of lead-based HPs, solar cell design, challenges and pitfalls on the way to the HP cells competitive with the silicon counterparts, as well as issues of stability, environmental impact and possible recycling of the Pb-HP-based devices [11–13,16,23,26–27,29–50]. A tremendous amount of work has been performed in searching for the most efficient and stable compounds with mixed cations (e.g., MA^+^/Cs^+^, MA^+^/FA^+^) and mixed halide components [20–21,29–30,49,51].

It was recognized that Pb-HPs, especially with inorganic Cs^+^ ions that have no asymmetry typical for organic MA or FA cations, also have an extremely high potential for application in other areas, in particular as light emitters for LEDs, laser applications and in photodetectors [28–31,35,43,47,50,52–54]. The versatility of the possible optoelectronic applications of Pb-HPs has stimulated an explosive progress in preparative chemistry and photophysics of HP nanocrystals (NCs) [16,24,28–30,43,50,53–56]. Recently, broad recognition was gained by 1D and 2D layered hybrid perovskite materials with strong anisotropy of electron properties. Such materials are currently treated as very promising light harvesters with highly tunable optical and charge transport characteristics [14,16,24,28–31,44,47,57–58].

In recent years, some saturation of the initial drastic growth of the power conversion efficiency (PCE) of lead-HP-based solar cells has been observed (Figure 1a). Simultaneously a number of critical challenges related to these materials were recognized as limiting their future broad implementation [29–31,36,39–40,42,48–50,57,59]. The unrivaled light-to-current conversion efficiency of lead-based HP absorbers is largely compromised by the hydrolytic and photochemical instability of Pb-HPs as well as the highly toxic character of the released Pb^2+^, which requires the development of special recycling protocols [40,42]. While the first problem seems to be solvable, in particular by the encapsulation and a design of the cationic sublattice, the presence of Pb^2+^ cannot be avoided.

Lead is allowed for usage in the outdoor photovoltaic modules, but the utilization of alternative, less toxic metals is highly welcomed [40,42,54,57,59–61]. One of the promising routes to decrease the environmental burden of Pb-HP cells and to maintain reasonably high PCEs was to partially substitute Pb^2+^ with other double-charged cations, such as Sn^2+^, Mn^2+^, or Ge^2+^, where the tin-based materials have gained the most attention and progress [10,16,18,29–30,38,44,54,59–65]. The Sn-based HPs (CsSnX_3_, MASnX_3_) show a high charge carrier mobility and diffusion length, comparable to the Pb-based analogs [16,18,57,59–60,62–63,65–66]. Despite large recombination losses reported for CsSnX_3_ materials, solar cells based on these compounds showed a promising light conversion efficiency of ≈13% indicating a great potential for the lead-free HP [59,62–63]. Numerous attempts and probes have shown that photoactive perovskite compounds can be developed also for other metals, in particular for Bi^3+^, Sb^3+^, Cu^2+^, and combinations of Ag^+^/Bi^3+^, Ag^+^/Sb^3+^, and In^+^/Bi^3+^.

A drastic growth of interest in lead-free HPs has been witnessed in the past three years, where the number of relevant publications has skyrocketed by more than an order of magnitude from 2014 to 2017 with the number of papers published in the first half of 2018 already exceeding the number in 2016 (Figure 1b). The fast progress in the area of lead-free HPs is also supported by an ever-growing number of review papers trying to distinguish the most promising venues and materials and to suggest outlines of further exploration [28–30,42,50,53–54,57,59–61,64–65].

**Figure 2:**
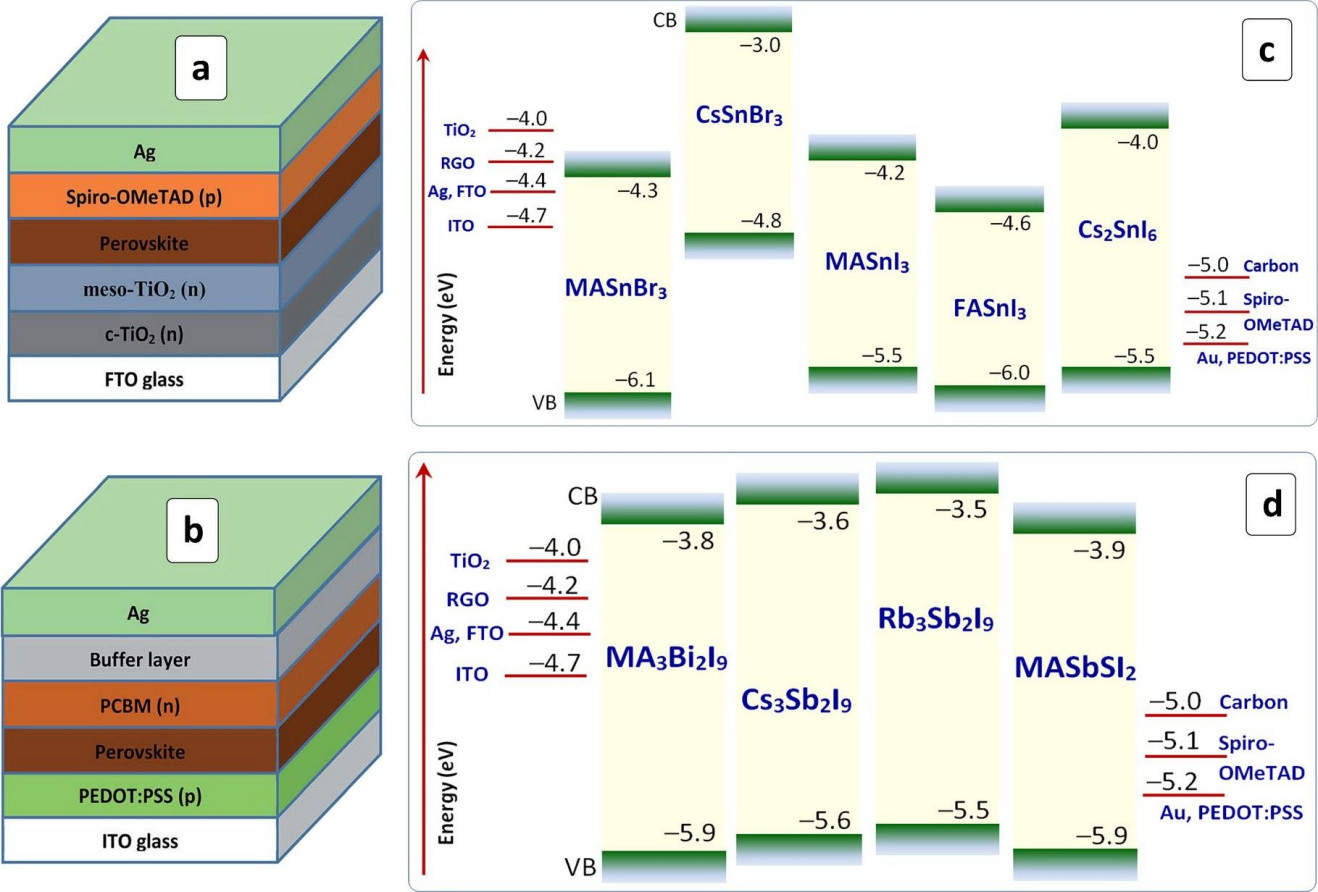
Schemes of conventional (a) and inverted (b) HP-based solar cell; energy diagrams of selected Sn-based HPs (c) and Bi- and Sb-based HPs (d) relative to the levels of some ETL and HTL materials. The CB/VB levels are taken from [78] (MASnBr_3_), [79] (CsSnBr_3_), [80–81] (MASnI_3_), [80,82] (FASnI_3_), and [80,83] (Cs_2_SnI_6_), [67–86] (MA_3_Bi_2_I_9_), [87] (MASbSI_2_), [88–90] (Cs_3_Sb_2_I_9_), and [91] (Rb_3_Sb_2_I_9_). (a,b) Reprinted with permission from [27], copyright 2018 The Royal Society of Chemistry.

Historically, the first HP cell was built basing on a “classic” design of the dye-sensitized solar cells with the HP layer acting as a sensitizer of a mesoporous metal oxide (TiO_2_) scaffold [67]. Later, it was recognized that Pb-based HPs are incomparably more efficient when applied as light harvesters in photovoltaic planar or bulk heterojunction solar cells. Such cells have two designs – a “conventional” n–i–p design with a HP layer deposited onto the metal oxide electron transport layer (ETL) and then covered with an organic hole transport layer (HTL) and an “inverted” p–i–n design, where an HP layer is formed on an HTL support and covered with an organic ETL, such as fullerene derivatives (see below in Figure 2). The conventional n–i–p scheme dominates in the studies of HPs with the typical ETLs being titania and various TiO_2_-based composites [27]. The most efficient and frequently used HTLs are among the derivatives of spirobifluorene (Spiro-OMeTAD, see Table 1) and polythiophenes (PEDOT:PSS).

**Table 1:** Photovoltaic characteristics of selected mixed and lead-free HP-based solar cells.^a^

Perovskite	Cell configuration	*J*_sc_, A/cm^2^	*V*_oc_, V	FF	PCE, %	Ref.

**Sn,Pb-HPs**						

MASn_0.5_Pb_0.5_I_3_	FTP/TiO_2_/**HP**/P3HT/Au-Ag	20.04	0.42	0.50	4.18	[68]
MASn_0.1_Pb_0.9_IBr_2_	FTO/TiO_2_/**HP**/C	14.3	1.26	0.63	11.33	[69]
MAPb_0.5_Sn_0.5_(I_0.8_Br_0.2_)_3_	ITO/PEDOT:PSS/**HP**/ICBA/Ag	25.9	0.90	0.75	17.63	[70]

**Sn-HPs**						

MASnI_3_	FTO/TiO_2_/**HP**/PTAA/Au	24.28	0.429	0.64	6.63	[71]
CsSnI_3_	ITO/TiO_2_/HP/Spiro-MeOTAD/Au	23.2	0.86	0.65	12.96	[72]
MASnIBr_1.8_Cl_0.2_	FTO/TiO_2_/**HP**/C	14.0	0.38	0.57	3.1	[73]
MASnIBr_2_	FTO/TiO_2_/**HP**/Spiro-MeOTAD/Au	12.30	0.82	0.57	5.73	[74]
BA_2_MA_3_Sn_4_I_13_	FTO/TiO_2_/**HP**/PTAA/Au	24.1	0.229	0.46	2.53	[75]
FASnI_3_	ITO/PEDOT/**HP**/C_60_/BCP/Al	24.1	0.525	0.71	9.0	[76]
FASnI_3_	ITO/SnO_2_/C_60_/HP/Spiro-MeOTAD/Ag	22.45	0.47	0.68	7.09	[77]

**Ge-HPs**						

MAGeI_3_	FTO/TiO_2_/**HP**/Spiro-MeOTAD/Au	4.0	0.150	0.30	0.20	[119]
MAGeI_2.7_Br_0.3_	ITO/PEDOT:PSS/**HP**/PC_70_BM/Ag	2.43	0.460	0.51	0.57	[123]

**Bi-HPs**						

MA_3_Bi_2_I_9_	FTO/TiO_2_/**HP**/Spiro-MeOTAD/Au	0.798	0.486	0.42	0.164	[132]
MA_3_Bi_2_I_9_	FTO/TiO_2_/**HP**/Spiro-MeOTAD/Au	3.00	0.83	0.79	1.64	[64]
MA_3_Bi_2_I_9_	FTO/TiO_2_/**HP**/P3HT/Au	1.157	0.354	0.464	0.19	[65]
Cs_2_AgBiBr_6_	FTO/TiO_2_/**HP**/Spiro-MeOTAD/Au	3.93	0.98	0.63	2.43	[170]

**Sb-HPs**						

MA_3_Sb_2_I_9_	ITO/PEDOT:PSS/**HP**/PC_61_BM/ZnO/Al	1.0	0.896	0.55	0.49	[130]
MA_3_Sb_2_I_9_	ITO/PEDOT:PSS/**HP**/PC_71_BM/C_60_-BCP/Al	5.41	0.62	0.61	2.04	[70]
MASbSI_2_	FTO/TiO_2_/**HP**/PCPSTBT	8.12	0.65	0.59	3.08	[67]
MA_3_Sb_2_Cl*_x_*I_9−_*_x_*	FTO/TiO_2_/**HP**/Spiro-MeOTAD/Au	5.04	0.69	0.63	2.19	[131]
Rb_3_Sb_2_I_9_	FTO/TiO_2_/**HP**/Poly-TPD/Au	2.11	0.55	0.57	0.66	[71]
Cs_3_Sb_2_I_9_	ITO/PEDOT:PSS/**HP**/PC_70_BM/Al	5.31	0.72	0.39	1.49	[69]

^a^The accuracy of values presented as reported, *J*_sc_ – short-circuit photocurrent density, *V*_oc_ – open-circuit photovoltage, FF – fill factor. Abbreviations: BA = CH_3_(CH_2_)_3_NH_3_; P3HT – poly(3-hexylthiophen-2,5-diyl); Spiro-MeOTAD – 2,2′,7,7′-tetrakis-(*N*,*N*-di-*p*-methoxyphenylamine)-9,9′-spirobifluorene; PCPSTBT –poly(2,6-(4,4-bis(2-ethylhexyl)-4*H*-cyclopenta[2,1-*b*;3,4-*b*′]dithiophene)-alt-4,7(2,1,3-benzothiadiazole)); poly-TPD – poly(*N*,*N*'-bis-4-butylphenyl-*N*,*N*'-bisphenyl)benzidine; PEDOT:PSS – poly(ethylenedioxythiophene):polystyrenesulfonate; PC_x_BM – [6,6]-phenyl C_x_ butyric acid methyl ester; BCP – 2,9-dimethyl-4,7-diphenyl-1,10-phenanthroline; ICBA – indene–C_60_ adduct; PTAA – poly[bis(4-phenyl)(2,4,6-trimethylphenyl)-amine].

Recently, very good prospects were recognized for the cell design without organic HTL and back contacts, both roles played by a carbon layer. Panels (c) and (d) in Figure 2 show CB/VB levels of selected lead-free perovskites based on Sn^2+^/Sn^4+^ (Figure 2c) and Sb^3+^/Bi^3+^ (Figure 2d) with respect to the acceptor/donor levels of a series of typical ETL/HTL materials.

The present review aims to survey lead-free perovskites and closely related compounds reported in light of their possible applications as light harvesters in photovoltaic systems. Special focus is placed on the influence of the spatial organization of HP materials both on the micro- and nanoscale levels with respect to the performance and stability of perovskite-based solar light converters. The survey starts from HPs with lead ions partially substituted by isovalent cations of other less toxic metals, then focuses on lead-free HPs where the central metal ion is in the oxidation state of +2, predominantly, Sn^2+^ and Ge^2+^. Then, hybrid perovskite materials formed on the basis of M^3+^ cations (Sb^3+^, Bi^3+^) as well as on combinations of M^+^/M^3+^ ions aliovalent to 2Pb^2+^ (like Ag^+^/Bi^3+^, Ag^+^/Sb^3+^) are discussed as one of the most promising avenues to further progress in the research of lead-free perovskite light harvesters. The final Conclusion and Outlook section is focused on future strategies of the design of photovoltaic systems on lead-free perovskite compounds and the materials that have a high potential to be discovered.

### Hybrid perovskites with partially/completely substituted Pb^2+^ cations

#### Hybrid perovskites with partially substituted lead ions

Using a small “tool kit” of two metals, Sn and Pb, and two organic cations, A = MA and FA, a broad variety of isostructural Pb-, Sn- and Pb–Sn-based ASn*_x_*Pb_1−_*_x_*I_3_ HPs can be synthesized with a bandgap varying from 1.25 to 1.75 eV depending on the HP composition [92]. By simultaneously tuning the composition of Pb–Sn and halide components, a solar light absorber was designed with a bandgap of 1.35 eV ideal for the solar light harvesting. The inverted cells based on MAPb_0.5_Sn_0.5_(I_0.8_Br_0.2_)_3_ demonstrated PCEs of up to 17.63% [70]. A suppressed lattice disorder of this HP results in a low density of traps and sub-bandgap states reflecting in a relatively small *E*_g_–*V*_oc_ loss of 0.45 eV [70].

The bandgap of alloyed FASn*_x_*Pb_1−_*_x_*I_3_ HPs was found to vary in an unexpected way, that is, decreasing upon the introduction of Sn from ≈1.5 eV for MAPI to 1.24 eV for *x* = 0.4 and then increasing to ≈1.3 eV for the FASnI_3_ perovskite [93]. The “bowing” of the *E*_g_(*x*) dependence may originate from a transition from cubic to orthorhombic lattice symmetry upon increasing tin content [93]. A similar anomalous variation of the bandgap, as well as the CB/VB level positions with the Sn content, was reported for CH_3_NH_3_Sn*_x_*Pb_1−_*_x_*I_3−_*_y_*Cl*_y_* HPs [94].

A study of alloyed ASn_1−_*_x_*Pb*_x_*I_3_ (A = Cs^+^, FA^+^, MA^+^ or their combinations) produced in the form of NCs showed the mixed compounds to be much more stable to ambient air as compared to both ASnI_3_ and APbI_3_ individually [95–97]. The cation-exchange approach applied to produce FASn_1−_*_x_*Pb*_x_*I_3_ and FAPbI_3_ from FASnI_3_ is expected to be a general one and appropriate for the introduction of other isovalent and aliovalent cations such as Mn^2+^, Co^2+^, Bi^3+^, and Al^3+^ into the sites of Sn^2+^ or Pb^2+^ [97]. The chemical stability of mixed Sn–Pb perovskites can be further enhanced by the passivation with a C_60_ layer [98]. The fullerene was found to eliminate the surface trap states of MAPb_0.75_Sn_0.25_I_3_ crystals suppressing the electron–hole recombination as well as shielding the HP layer from the ambient moisture and oxygen.

**Figure 3:**
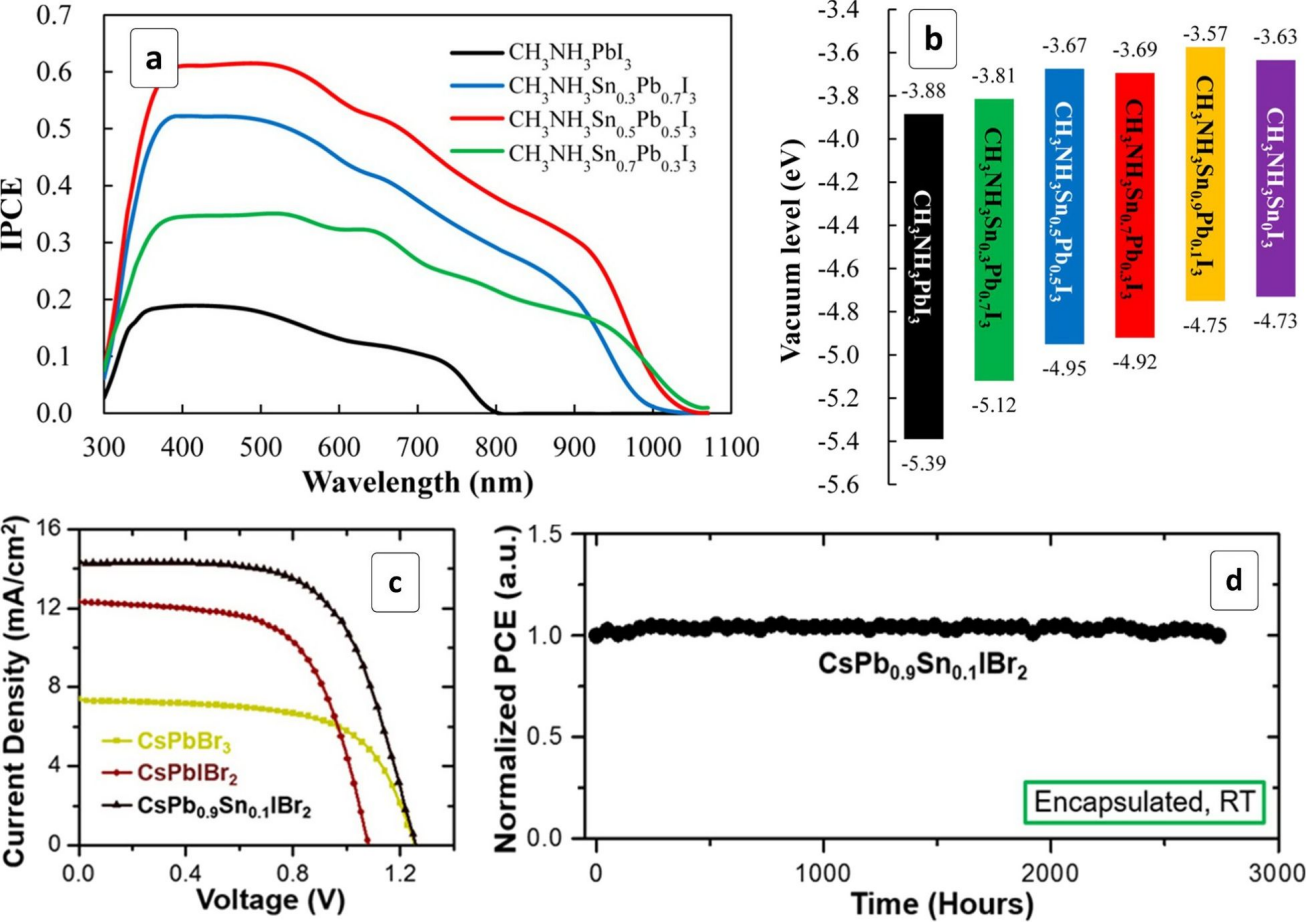
(a,b) Internal photon-to-current conversion efficiency (IPCE) spectra of solar cells comprising a series of Sn,Pb-based HPs with different Sn/Pb ratios (a) and energy diagram of the corresponding perovskites (b); (c) current–voltage curves for solar cells based on CsPbBr_3_, CsPbIBr_2_ and CsPb_0.9_Sn_0.1_IBr_2_ HPs; (d) test of prolonged performance stability of a solar cell based on CsPb_0.9_Sn_0.1_IBr_2_ HP, RT – room temperature. (a,b) Reprinted with permission from [68], copyright 2014 American Chemical Society and (c,d) Reprinted with permission from [69], copyright 2017 American Chemical Society.

Solar cells with mixed MASn*_x_*Pb_1−_*_x_*I_3_ HPs and a P3HT HTL showed a dome-shaped PCE dependence on the lead content (Figure 3a), where a maximum η of 4.18% was achieved at an Sn/Pb atomic ratio of 1:1 [68]. The conventional MAPI HP displayed a much lower efficiency, while a pure Sn-based HP turned out to be inactive with this HTL material. A higher efficiency of mixed Sn,Pb-HPs stems largely from a broader absorption range extending to λ_e_ = 1060 nm as compared to only 800 nm for MAPI (Figure 3a). A partial substitution of the lead with tin was found to affect the VB position of the HP much stronger than the corresponding CB level (Figure 3b). This fact hints at the importance of the selection of an appropriate hole transport material for each particular HP composition to realize the potential of such materials to a full extent.

The introduction of 10% Sn into CsPbIBr_2_ HP results in a bandgap narrowing from 1.90 to 1.79 eV and an increase of the solar cell performance (Figure 3c) from 8.25% for the undoped Pb-HP to 11.33% for the CsSn_0.1_Pb_0.9_IBr_2_-based device [69]. The latter cell also exhibited a record *V*_oc_ of 1.26 V amounting to ≈70% of the optical bandgap and vividly showing a high potential of such photovoltaic materials. Additionally, the cell fitted with an encapsulating protective layer showed a remarkable stability with the PCE unchanged in a more than 2500 h test trial (Figure 3d) [69].

Manganese(II) ions were found to substitute Pb(II) in MA-Pb-Cl-Br HPs, the perovskite preserving the crystal structure up to 90% Mn [99]. Up to 46% Pb^2+^ ions can be exchanged with Mn^2+^ in CsPbCl_3_ NCs produced by hot-injection, resulting in a highly increased photoluminescence (PL) efficiency [100]. Mixed MAPb*_x_*Mn_1−_*_x_*I_1+2_*_x_*Cl_2−2_*_x_* (*x* = 0.1–1.0) synthesized by a solid-state reaction displayed an unprecedentedly high open-circuit voltage of up to 1.19 V and fill factor (FF) of almost 90% when introduced into inverted solar cells with PEDOT:PSS and PCBM charge transport layers [101]. Despite the high *V*_oc_ and FF values, the cells showed quite a low efficiency of ≈0.3% indicating huge recombinational losses and leaving large room for further improvement of the structural quality of the perovskite absorber layer.

The substitution of a mere 2% lead with Sr(II) in CsPbI_2_Br HP was found to result in a spectacular PCE increase from 6.6% to 10.1% and an enhancement of the thermal HP stability [102]. Strontium ions accumulate in a surface layer of the HP film exerting a passivating effect and resulting in a longer charge carrier lifetime [102]. The introduction of Ca^2+^ on the Pb^2+^ sites of CsPbI_3_ HP results in a more homogeneous a better contact between the HP and HTL, as well as the surface passivation by a Ca-enriched surface layer [103]. The best Ca-substituted CsPbI_3_ HPs show a PCE of higher than 13% and maintain more than 85% of the initial efficiency for more than two months of testing with encapsulation [103].

A partial substitution of Pb(II) with In(III) yields HPs with a reduced lead content and a promising PCE exceeding 17.5% [104]. The introduction of Sb(III) during the growth of MAPI HP results in the substitution of lead with antimony and formation of a MA_3_Sb_2_I_9_ layer on the surface of growing MAPI crystals thus limiting their size to ≈50 nm [105]. An enhanced PL of the Sb-doped MAPI crystals indicates that the electron–hole recombination is efficiently suppressed by the surface antimony-rich layer [105].

#### Sn-based hybrid perovskites

The MASnI_3_ perovskite displays a bandgap of ≈1.3 eV [74,81] corresponding to the absorption onset at λ_e_ ≈ 950 nm, which is significantly shifted as compared to the MAPI counterpart (*E*_g_ = 1.55 eV, ≈800 nm) [74] and comparably high absorption coefficients of ≈10^5^ cm^−1^ [106]. Thick MASnI_3_ perovskite wafers synthesized by a temperature-reduction-induced crystallization showed an even narrower bandgap of ≈1.2 eV [107]. The FASnI_3_ compound with a bulkier formamidinium cation displayed a larger bandgap of 1.41 eV [82]. A partial substitution of I with Br results in a controlled expansion of the bandgap up to 1.68 eV for FASnI_2_Br [108]. The reported bandgaps of selected Sn-based HPs are collected in Table 2.

**Table 2:** Bandgap and approximate absorption band edge position (λ_e_) of selected Sn-based hybrid perovskites.

Perovskite	*E*_g_, eV	λ_e_, nm	Ref.

MASnCl_3_	2.1	590	[109]
MASnBr_3_	2.2	570	[78]
CsSnBr_3_	1.80	690	[110]
MASnI_3_	1.21–1.23 1.3	1010–1030 960	[111] [74,79,81]
FASnI_3_	1.41	880	[82]
FASnI_2_Br	1.68	740	[108]
Cs_2_SnI_6_	1.48	840	[83,112]
MA_2_SnI_6_	1.81	690	[54]

Similar to MAPI, the morphology of MASnI_3_ plays an important role in the efficiency of the solar cells based on this light harvester. This fact stimulated a screening of suitable solvents and deposition conditions, revealing dimethylsulfoxide (DMSO) as one of the most promising candidates for spin-coating deposition technology [113–114], which produced uniform pin-hole-free MASnI_3_ films due to the formation of an intermediate SnI_2_×DMSO phase [114]. Trimethylamine acts in a similar way forming intermediate complexes with SnI_2_ (and SnF_2_ as a stability-enhancing additive) and facilitating the formation of dense and uniform MASnI_3_ and FASnI_3_ films [77]. The simultaneous presence of the trimethylamine and SnF_2_ was found to be crucial both for conventional and inverted solar cell configurations. For example, the modification of an inverted FASnI_3_-based cell with SnF_2_ resulted in a spectacular PCE increase from 0.52 to 4.20% with a further increase to 7.09% (Table 1) induced by the introduction of trimethylamine as a morphology-directing agent [77].

**Figure 4:**
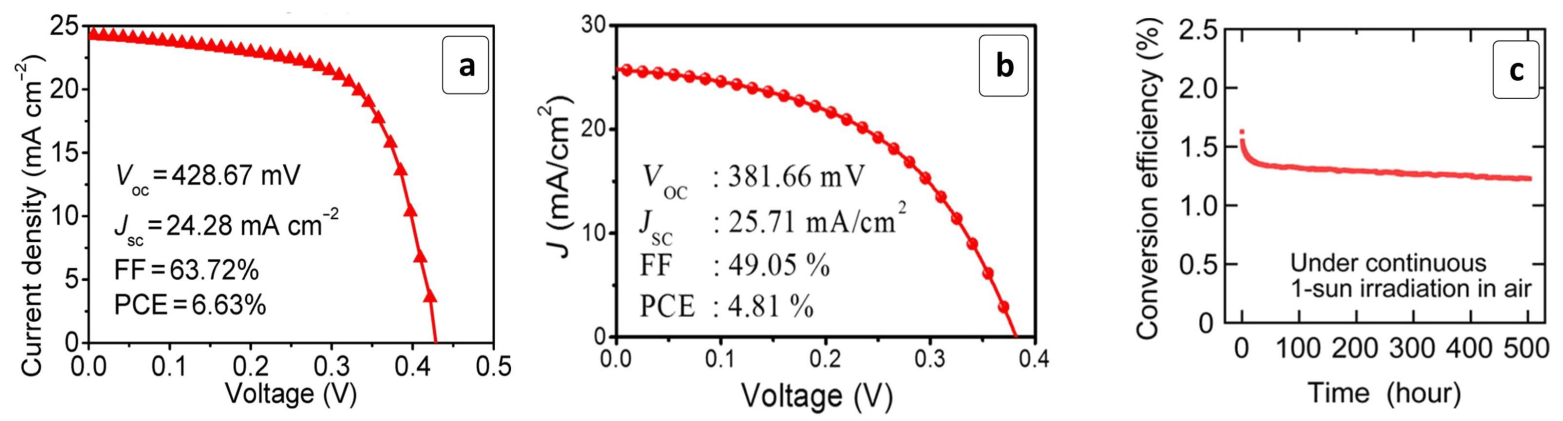
(a,b) Photocurrent density–voltage curves recorded for the solar cells based on MASnI_3_ HPs (a) and CsSnI_3_ HPs (b); (c) test of prolonged performance stability of a solar cell based on MASnI_3_ HPs. (a) Reprinted with permission from [71], copyright 2017 American Chemical Society; (b) Reprinted with permission from [80], copyright 2017 American Chemical Society; (c) Reprinted with permission from [118], copyright 2017 American Chemical Society.

The quality of MASnI_3_ films as components of solar cells can be ameliorated by introducing ethylenediamine (en) acting simultaneously as an additional organic cation in the HP lattice and as a morphology-directing agent [71]. The cells with such modified MASnI_3_ absorbers showed a PCE of 6.63% (Table 1) with a relatively high FF of ≈64% (Figure 4a). High-quality MASnI_3_ films yielding a PCE of 1.86% were prepared using a low-temperature vapor-assisted deposition [115].

The stability of MASnI_3_- and CsSnI_3_-based solar cells is largely compromised by a low HP stability to oxidation [80,116]. It was found that the Sn^2+^ state of the central ion can be stabilized by introducing an excess of SnI_2_, with the best cells showing a PCE of 4.81% (Figure 4b) and a prolonged stability of the photovoltaic parameters [80]. Calculations by the density functional theory (DFT) indicated that a partial substitution of Cs^+^ with Rb^+^ should considerably increase the stability of CsSnI_3_ [117].

**Figure 5:**
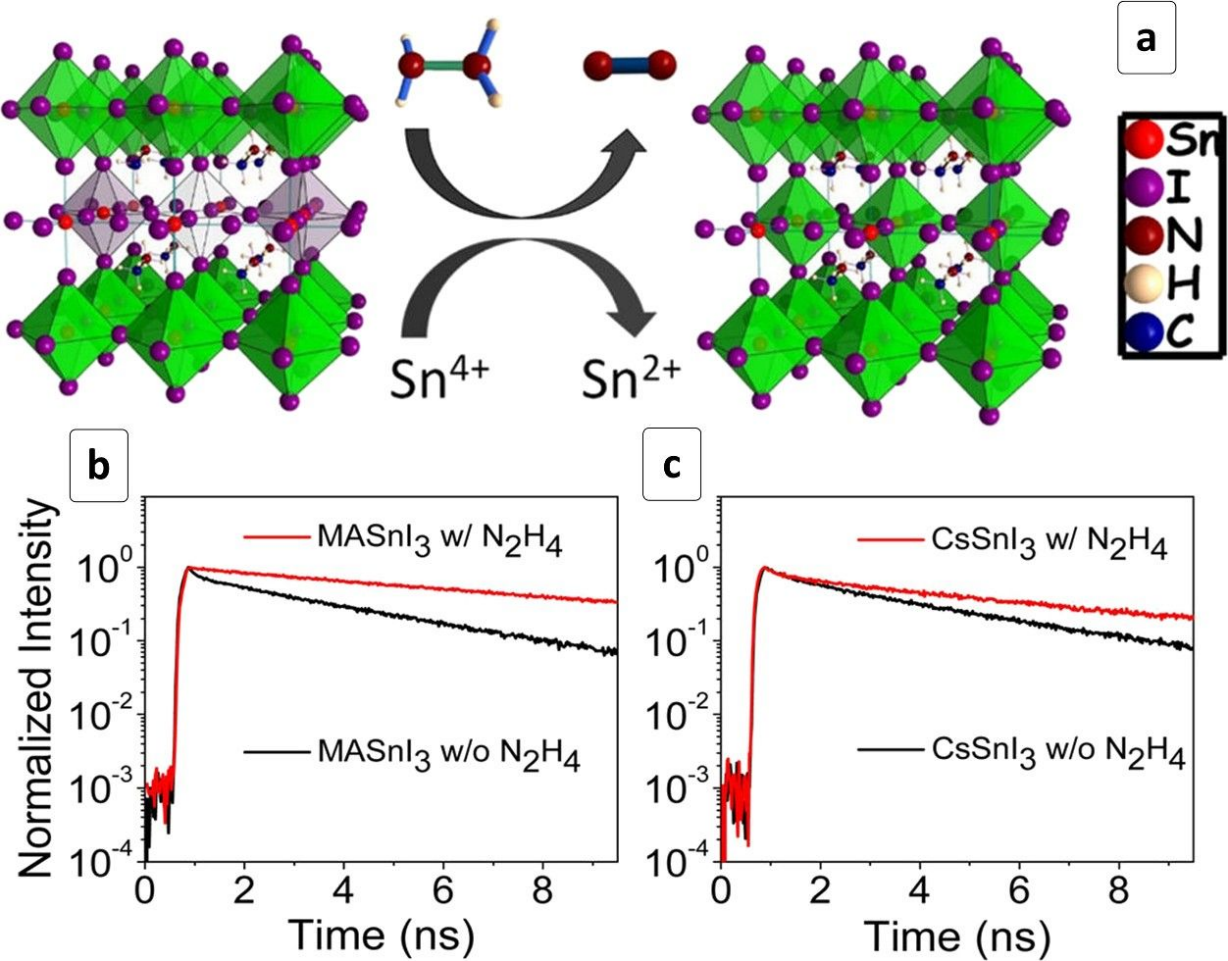
(a) Scheme of a possible mechanism of Sn-based HP transformation upon reaction with hydrazine; (b,c) kinetic photoluminescence decay curves for MASnI_3_ (b) and CsSnI_3_ (c) produced without treatment (black lines) and with an N_2_H_4_ vapor treatment (red lines). Reprinted and adapted with permission from [119], copyright, 2016 American Chemical Society.

To avoid a partial conversion of Sn^2+^ into Sn^4+^, the latter acting as charge carrier traps in ASnX_3_ HPs, it was suggested to deposit the perovskite layer under a reductive atmosphere, for example, in the presence of hydrazine vapors [119]. The conversion of Sn^4+^ into Sn^2+^, which can be described as 2SnI_6_^2–^ + N_2_H_4_ = 2SnI_4_^2–^ + N_2_ + 2HI, results in a reduction of the density of Sn^2+^ vacancies (Figure 5a) suppressing the undesirable p-type conductivity and reverting the perovskites back to n-type semiconducting behavior [119]. The hydrazine treatment results in an appreciable increase of the radiative lifetime of ASnX_3_ HPs irrespective of the type of cation A (Figure 5b,c) and halide component composition, clearly indicating a reduction of the trap-mediated non-radiative recombination losses.

The effect of elimination of Sn^2+^ vacancies in FASnI_3_ HP resulting from tin(II) oxidation can also be achieved by a partial substitution of iodide with bromide [120]. The devices with a mixed Br/I halide component displayed a reduced dark current and lower recombination rate, resulting in an increased *V*_oc_ and FF and showed a PCE above 5%, whereby the cells retained stability over a 1000 h time trial span [120]. In a similar manner, the mixed CsSnIBr_2_ HP revealed a higher stability and a lower density of Sn^2+^ vacancies which can be further decreased by growing the HP crystals in the presence of hypophosphoric acid as a tin(II) complexant [121]. The corresponding cells revealed the stable PCE for a 77 day trial at room temperature and during a 9 h test at 473 K [121].

An elegant way of simultaneously ordering MASnI_3_ deposits and protecting them from exposure to air/moisture was suggested via the growth of HP nanowires in the pores of anodized alumina membranes [122]. The effective blockage of the diffusion of water and oxygen molecules to the alumina-incorporated MASnI_3_ nanowires resulted in a three order of magnitude slower degradation of this material as compared to planar films of the same composition [122].

It was found that of the three “homologs” of CsSnX_3_ HPs (X = Cl, Br, I) the bromide-based compound shows an exceptionally high photochemical and chemical stability, which can further be enhanced by doping with SnF_2_ [110].

The stability of MASnI_3_-based cells can also be strongly enhanced by doping with SnF_2_ [118,123–124]. The doped materials showed a remarkable stability when illuminated under ambient air conditions without additional encapsulation (Figure 4c). Additionally, the SnF_2_ doping results in a decrease of the HP bandgap down to 1.25 eV, which is highly beneficial for the cell performance. This redshift effect was attributed to the Burstein–Moss effect arising from a significant doping of the absorber material with holes [118]. The SnF_2_ doping was found to almost double the radiative lifetime of charge carriers and considerably increase the carrier diffusion length [125]. A similar approach can be applied to increase the stability of FASnI_3_ absorbers [82].

The quality and stability of FASnI_3_ can be strongly enhanced by introducing trace amounts of 2D tin HPs comprising both FA and 2-phenylethylammonium cations [126]. The presence of 2D HP favors a more homogeneous growth of FASnI_3_ crystals, resulting in a reduced number of grain boundaries and the suppression of the formation of Sn^4+^. The high quality of such FASnI_3_ films was evidenced by a strongly reduced background carrier density and a longer charge carrier lifetime [126]. The solar cells produced from these highly uniform HP layers revealed a negligible hysteresis and no light soaking effect, indicating a largely suppressed recombination. The best PCE was 9%, which is by 50% higher than for similar cells with the HP layer modified by SnF_2_ [76].

**Figure 6:**
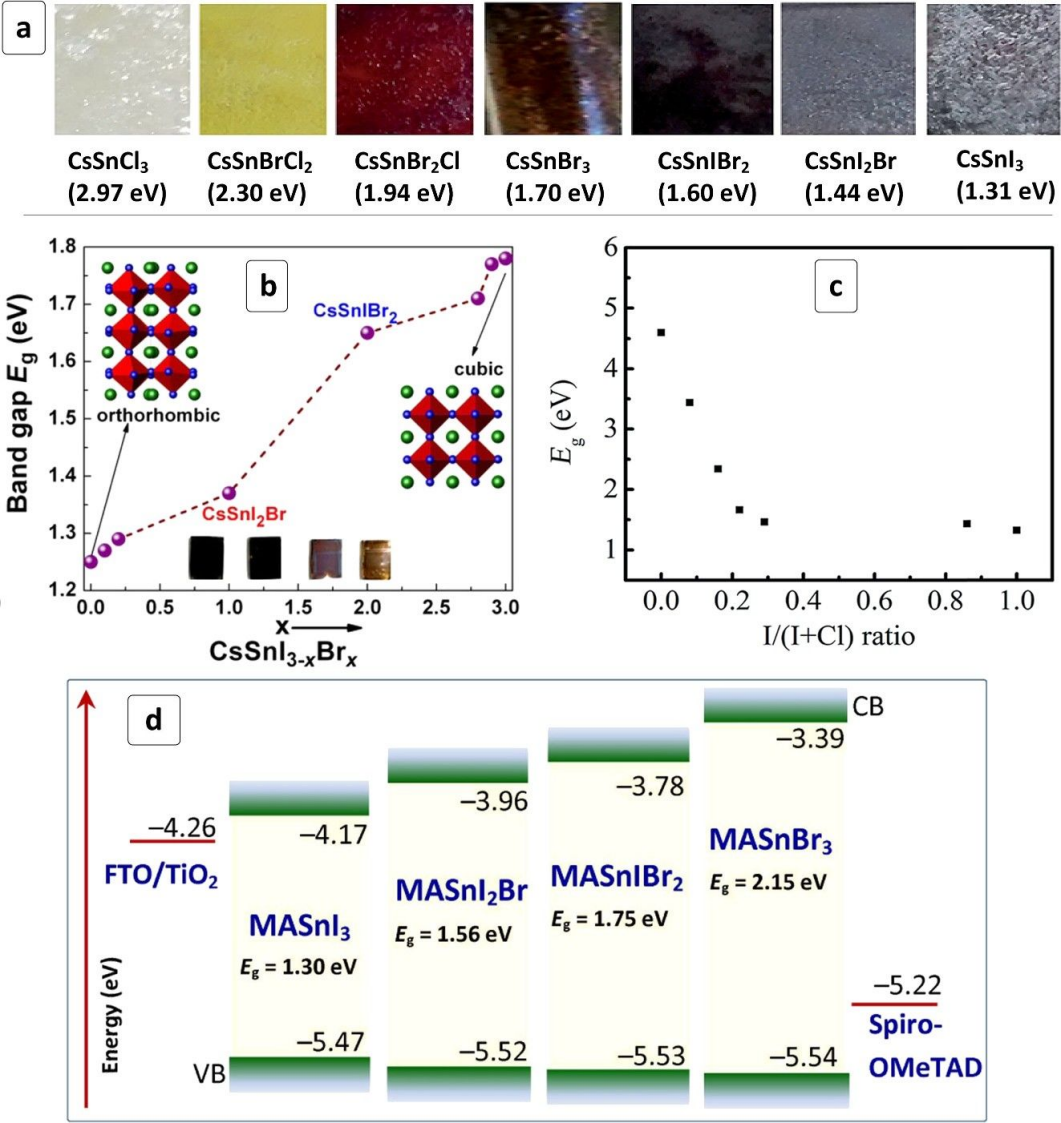
(a) Photographs of HP films produced from different CsSnI_3−_*_x_*Br*_x_* and CsSnBr_3−_*_x_*Cl_3_ compounds (bandgaps are provided in parenthesis); (b,c) Bandgap as a function of the composition of CsSnI_3−_*_x_*Br*_x_* HPs (b) and Cs_2_SnI*_x_*Cl_6−_*_x_* HPs (c); (d) energy diagrams of solar cells based on MASnI_3−_*_x_*Br*_x_* HPs, FTO/TiO_2_ ETL and Spiro-OMeTAD HTL. The diagram is plotted using numerical data reported in [74]. (a) Reprinted and adapted from [126], copyright 2016 The Royal Society of Chemistry; (b) Reprinted and adapted from [127], copyright 2015 American Chemical Society; (c) Reprinted and adapted from [128], copyright 2018 The Royal Society of Chemistry.

A compositional variation of the halide component of CsSnX_3_ HPs is a potent instrument allowing the bandgap and CB/VB energies to be changed, and therefore, to affect the spectral sensitivity range and *V*_oc_ of the CsSnX_3_-based solar cells. The individual and mixed-halide Sn-HPs demonstrated a broad spectrum of bandgaps varying from 2.97 eV for CsSnCl_3_ to 1.31 eV for CsSnI_3_ with all possible intermediate values achievable by tailoring the type and relative content of halide ions (Figure 6a) [126].

By varying the bromide content in CsSnI_3−_*_x_*Br*_x_* perovskite the HP bandgap can be smoothly increased from 1.27 eV (CsSnI_3_) to 1.37 eV (CsSnI_2_Br) to 1.65 eV (CsSnIBr_2_) and up to 1.75 eV for CsSnBr_3_ (Figure 6b) [127]. The open-circuit voltage of the corresponding solar cells increases from ≈200 meV to 410 meV. A combination of two tendencies – a bandgap increase resulting in a narrowing of the spectral sensitivity range and a *V*_oc_ increase contributing to a higher PCE – results in an optimal HP composition of CsSnI_2_Br yielding the highest light harvesting efficiency [127].

Similar attempts of introducing chloride ions into Cs_2_SnI_6_ HP showed that single-phase compounds can exist only at compositions close to the individual I- and Cl-based compounds (Figure 6c), while at intermediate compositions a mixture of phases is typically produced [128]. In the case of I/Br-mixed Sn^4+^-based HP a series of single-phase Cs_2_SnI_6−_*_x_*Br*_x_* compounds were prepared with a bandgap tuned from 1.3 eV to 2.9 eV [129]. The highest PCE of 2.1% was reported for an intermediate composition corresponding to *x* = 2 [129].

Similar to the Cs-containing HPs, the optoelectronic properties of MASnX_3_ HPs can also be engineered by a partial substitution of halide anions. A gradual shift from MASnI_3_ to MASnBr_3_ via a series of intermediate solid-solution compounds (some illustrated by Figure 6d) results in an *E*_g_ expansion from 1.30 eV to 2.15 eV. Here the bandgap increment contributes mostly to a shift of the CB level to lower energies, while the VB level remains relatively unaffected [74]. For all compositions, the CB/VB positions are suitable for the construction of solar cells with a TiO_2_ ETL and Spiro-OMeTAD HTL. The CB shift results in an increase in the efficiency of electron transfer to the titania scaffold. This tendency is, however, counter-balanced by a reduction of the spectral sensitivity range due to an increased *E*_g_. Summarily, both trends result in the highest PCE observed for the intermediate MASnIBr_2_ HP (Table 1).

A combination of all three halides within a single tin-HP is a promising route to efficient and stable solar cell absorbers as shown on the example of MASnIBr_1.8_Cl_0.2_ HP displaying PCEs higher than 3% in a HTL-free cell as well as a long-term operational stability [73].

The light-harvesting MASnBr_3_ HP films were produced by evaporation of SnBr_2_ and MABr [78]. The co-evaporation technique was found to be preferable over a sequential deposition in terms of the photovoltaic efficiency due to the surface oxidation of the evaporated SnBr_2_ layer before the deposition of methylammonuim bromide [78].

The electron diffusion length in MASnI_3_ perovskite was estimated to be around 20 nm in contrast to over a micrometer in the corresponding lead HPs [111]. In view of this finding, the task of the preparation of large-as-possible HP grains with a minimized grain boundary area and surface defects seems to be of much lower significance for the Sn-based HPs, than for their lead-based counterparts. We can, therefore, expect a similar photovoltaic efficiency from micro- and nanometer Sn-HP crystals and try to affect the charge carrier transport through the HP/ETL and HP/HTL interfaces by tailoring the HP morphology on the nanometer scale. It was also found that the size-selected Cs_2_SnI_6_ NCs (12–49 nm) are characterized by a much smaller effective electron mass (0.12*m*_0_) as compared to the bulk HP (0.56*m*_0_) [130]. Therefore, one might expect a strong influence of the NC size on the CB level position favorable for the “band design” of the light absorber to fit the energy levels of various ETL materials.

**Figure 7:**
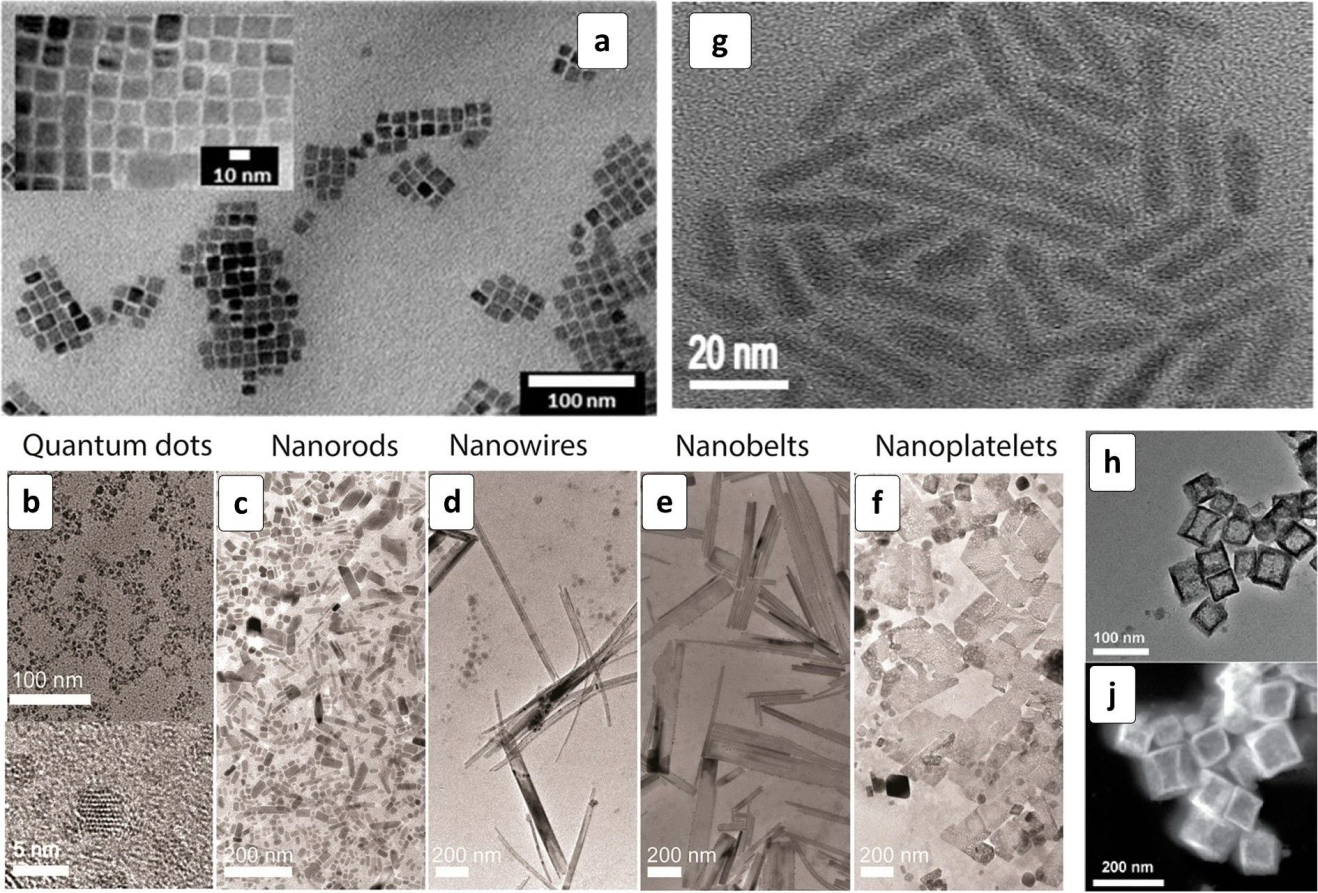
TEM (a–h) and STEM (j) images of CsSnI_3_ nanocrystals (NCs): (a) Cs_2_SnI_6_ in the form of NCs (b), nanorods (c,g), nanowires (d), nanobelts (e), and nanoplatelets (f); CsSnBr_3_ nanocages (h,j). (a) Reproduced with permission from [131], copyright 2016 American Chemical Society; (b–f) Reprinted and adapted with permission from [133], copyright 2016 American Chemical Society; (g) Reprinted and adapted with permission from [72], copyright 2016 American Chemical Society and (h,j) Reprinted and adapted with permission from [135], copyright 2017 American Chemical Society.

The Sn-based HPs can be synthesized in a variety of nanoscale morphologies, including 0D NCs, nanorods, nanoplatelets, etc., allowing possible size/shape effects to be investigated with respect to the optical, luminescent and photovoltaic properties of such materials. For example, reasonably monodisperse ≈10 nm NCs of a variety of CsSnX_3_ HPs (X = Cl, Br, I, Cl/Br, Br/I) can be produced (Figure 7a) by a general hot injection method using mildly reducing and coordinating tri-octylphosphine as a solvent for SnX_2_ [131]. A similar approach was recently applied for the synthesis of CsSnI_3_ nanoplates with a thickness of less than 4 nm [132]. The formation of CsSnX_3_ nanoscale phases requires the presence of toxic tri-octylphosphine, whereby the Sn(II)-based NCs are unstable and prone to oxidation in other high-boiling-point solvents [131–132].

Sn(IV)-based Cs_2_SnI_6_ HP was proposed as an alternative light absorber material that forms a variety of morphologies and can be synthesized in the more environmentally friendly oleic acid/oleylamine in octadecene [130,133–134]. The shape of nanoscale Cs_2_SnI_6_ can be tuned quite easily by varying the duration of crystal growth. The reaction between Sn(IV) oleate and CsI yields ≈2.5 nm NCs in a minute after cesium iodide injection (Figure 7b), where the NCs transform into HP nanorods after a 5 min ripening at 220 °C (Figure 7c) [133]. The Cs_2_SnI_6_ nanorods gradually transform into nanowires (Figure 7d) with the aspect ratio increasing from 3 to 28 after a 10 min reaction. At longer reaction times (30 min) nanowires transform into nanobelts (Figure 7e) that assemble into nanoplatelets with a thickness of ≈8 nm (Figure 7f) after a 60 min ripening at 220 °C.

When using Cs oleate as a precursor, the average size of the resulting Cs_2_SnI_6_ NCs can be smoothly varied from ≈12 to ≈40 nm by increasing the hot-injection synthesis temperature from 80 to 220 °C [130]. Such Cs_2_SnI_6_ NCs showed a size-dependent bandgap, decreasing from 1.47 nm for the smallest NCs to 1.36 eV to the largest ones. The NCs are free from any surface ligands and stable long enough for the preparation of solar cell electrodes [130].

The Cs_2_SnI_6_ HPs can be formed by in situ oxidizing unstable CsSnI_3_ with air oxygen [112] or directly deposited from a chemical bath [64]. The Cs_2_SnI_6_ perovskite is characterized by a bandgap of 1.48 eV and absorption coefficients over 10^5^ cm^−1^ above 1.7 eV [112].

MASnI_3_ and Cs_2_SnI_6_ HPs can be conveniently synthesized by the electro-assisted oxidation of Pb^0^ or Sn^0^ films (produced by evaporation) in alcohol solutions of alkali metal or alkyl ammonium halides [136]. The method is perfect for the direct formation of Sn-based HPs with tailored morphology and composition avoiding the use of unstable Sn^2+^/Sn^4+^ precursors and toxic solvents. Moreover, it was argued in [136] that the method allows for the thermodynamics-driven formation of HPs, resulting in a higher material quality and reproducibility as compared to the conventional kinetically quenched syntheses (solvent evaporation, spin or spray coating).

The shape control over CsSnX_3_ nanoscale phases grown in the presence of tri-octylphosphine oxide can be exerted by introducing complexants preferentially directing the NC growth along certain lattice planes. For example, CsSnX_3_ nanorods (X = Cl, Br, I) with a relatively homogeneous rod diameter distribution were synthesized in the presence of diethylenetriamine (Figure 7g) [72]. The CsSnX_3_ nanorods applied as light harvesters with TiO_2_/SpiroMeOTAD ETL/HTL combination revealed relatively high PCEs increasing from 9.66% for X = Cl to 10.46% for X = Br to 12.96% for X = I (Table 1), all three devices demonstrating a high *V*_oc_ of 0.85–0.87 V [72].

A controlled self-assembly phenomenon reported in [135] resulted in the formation of hollow “nanocages” composed of CsSnBr_3_ NCs (Figure 7h,j). The nanocages can be stabilized against decomposition caused by oxidation, hydrolysis or photochemical processes by a post-synthesis treatment with perfluorooctanoic acid.

**Figure 8:**
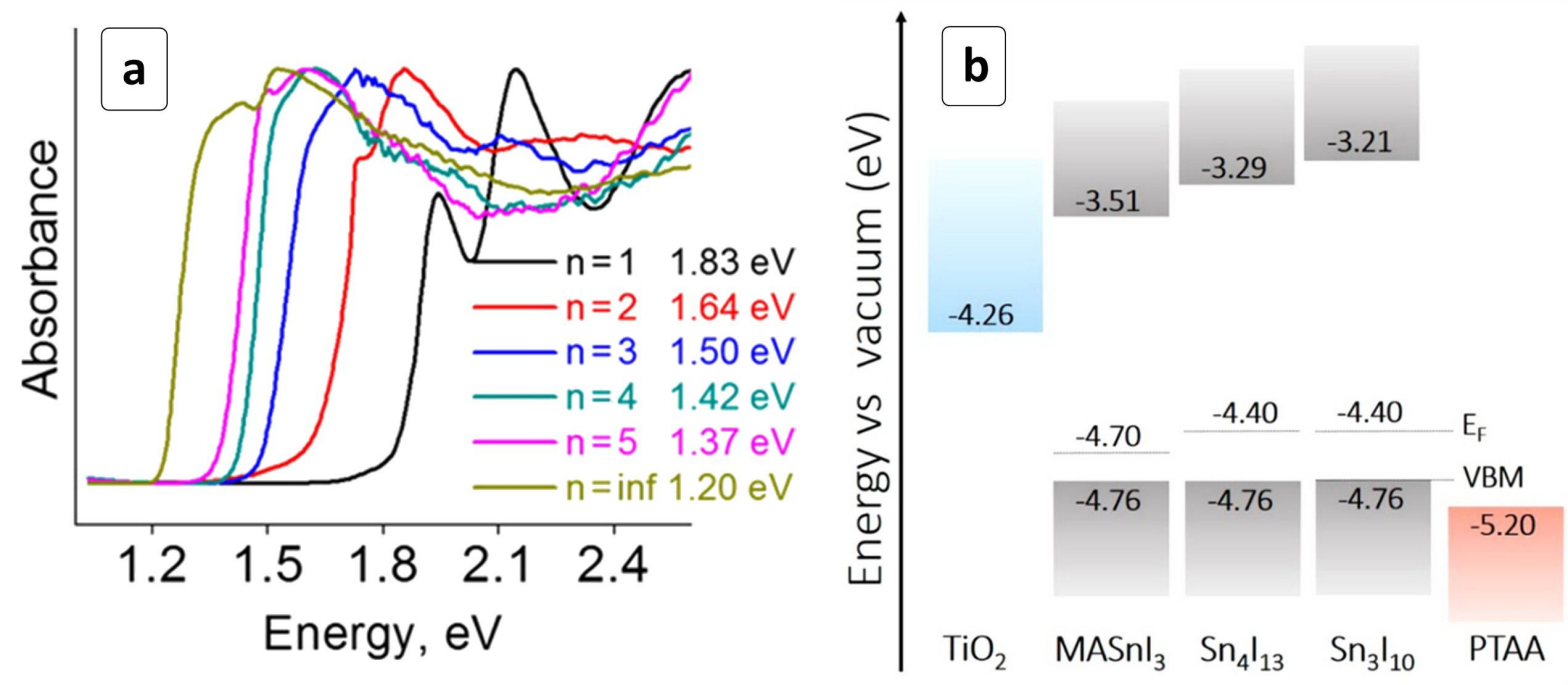
(a) Absorption spectra of (CH_3_(CH_2_)_3_NH_3_)_2_(CH_3_NH_3_)*_n_*_−1_Sn*_n_*I_3_*_n_*_+1_ homological HPs; (b) energy level alignment in solar cells with selected HPs. VBM is the valence band (VB) minimum, *E*_F_ is Fermi energy. Reprinted with permission from [75], copyright 2017 American Chemical Society.

A series of 2D (CH_3_(CH_2_)_3_NH_3_)_2_(CH_3_NH_3_)*_n_*_−1_Sn*_n_*I_3_*_n_*_+1_ perovskites was recently introduced as stable and promising alternatives of 3D ASnX_3_ HPs for photovoltaic applications [75]. The 2D HPs revealed semiconductor properties with a bandgap decreasing from 1.83 eV for *n* = 1 to 1.2 eV at *n* → ∞ (Figure 8a). The 2D HP layers can be selectively oriented parallel to the substrate when the HP is spin-coated from DMSO and perpendicular – if the deposition occurs from *N*,*N*-dimethylformamide (DMF). The CB energy position was found to strongly depend on the HP composition (Figure 8b).

This allows for the search of an optimum between the efficiency of the electron transfer to the TiO_2_ scaffold and the spectral sensitivity range defined by *E*_g_. The Sn_4_I_13_ “isomer” with a “close-to-ideal” *E*_g_ of 1.42 eV was suggested as an optimal light harvester, displaying a promising PCE of 2.5% (Table 1) [75].

Along with the photovoltaic cells with an HP layer sandwiched between ETL and HTL, the photo-electrochemical HP-based systems are explored as well, where an electron-shutting redox-couple is used for the charge exchange between the light-absorbing electrode and a counter electrode. For example, a solar cell based on a MASnI_3−_*_x_*Br*_x_* film coupled to a carbon counter electron by a dissolved benzoquinone redox-couple BQ^0^/BQ^−^ showed a PCE of 1.51% [137].

An FTO/TiO_2_/MASnCl_3_ photoanode (*E*_g_ = 2.1 eV) was combined with an FTO/Pt counter electrode and a solid/liquid electrolyte consisting of polyethylene oxide soaked with an acetonitrile solution of KI/I_2_ into a solar cell displaying PCEs of up to 0.55% [109].

The mixed CsSnI_2.95_F_0.05_ was successfully tested as an efficient HTL for dye-sensitized solar cells operating with PCEs of up to ≈10% [138].

#### Other M^2+^-based hybrid perovskites

**Figure 9:**
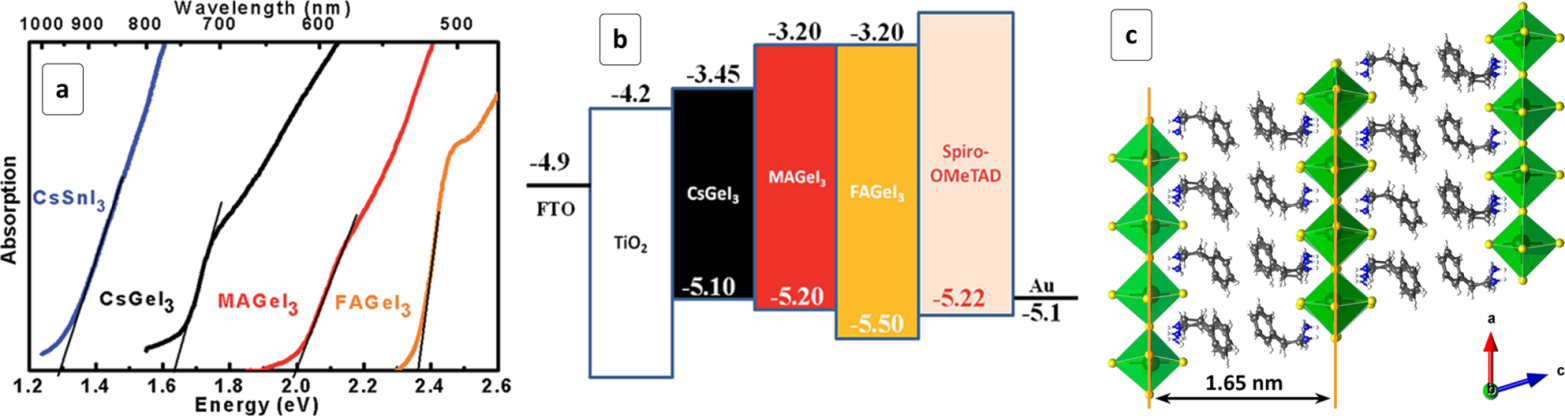
(a) Absorption spectra of AGeI_3_ HPs with different cations; (b) energy level alignment in solar cells with AGeI_3_ HPs; (c) structure of layered (PEA)_2_GeI_4_ HP. (a,b) Reprinted with permission from [140], copyright 2015 The Royal Society of Chemistry; (c) Reprinted with permission from [143], copyright 2017 American Chemical Society.

Germanium(II) forms a series of perovskites isostructural to MAPI with a bandgap decreasing from 3.76 eV for MAGeCl_3_ to 2.81 eV for MAGeBr_3_ to 1.61 eV for MAGeI_3_, the latter value close to *E*_g_ of MAPI (1.55 eV) [139]. Ge-based AGeI_3_ HPs with A = Cs^+^ (*E*_g_ = 1.63 eV), MA^+^ (2.0 eV), and FA^+^ (2.35 eV) were reported to be stable up to 150 °C but prone to the air oxidation [140]. The VB top and CB bottom of Ge-HPs are formed predominantly by Ge s- and p-orbitals, respectively, resulting in direct “intra-atomic”-like electron transitions. As the cation A size grows, the lattice constant increases, resulting in a further splitting between the Ge-related bonding and antibonding levels and, therefore, in an increase of the observed bandgap (Figure 9a) [140]. This behavior indicates favorable conditions for bandgap tuning by cationic substitutions. All three compounds have suitable CB/VB positions to be incorporated into the solar cells with typical ETL/HTL (Figure 9b) showing a PCE of 0.2% for MAGeI_3_-based cells (Table 1).

A DFT study of CsGeI_3_ HP showed that the iodide vacancy in this material can serve as a deep hole trap, in contrast to the corresponding Pb- and Sn-based HPs resulting in a reduction of the *V*_oc_ [141]. These results indicate that efforts should be applied for the synthesis of stoichiometric CsGeI_3_ materials as well as on the development of post-synthesis HI treatment of iodide-deficient CsGeI_3_ absorber layers.

Theoretical studies also showed a high susceptibility of the electron properties of MAGeI_3_ perovskite to the strain. Application of a compressive or dilating stress is expected to switch the HP conductivity from p- to n-type and vary the bandgap within a range of 1.35–2.50 eV [142].

Typically, Ge HPs are prone to hydrolytic decomposition when coming in contact with a humid environment [144]. Possible mechanisms and pathways of the hydrolytic degradation of MAGeI_3_ HP as a function of the crystal face were examined in detail in [145]. The stability of Ge-based HPs can be enhanced in mixed-halide HPs as well as by hydrophobic cations. In particular, by introducing cations that are more bulky than MA^+^, such as phenylethylamine (PEA) cation C_6_H_5_(CH_2_)_2_NH_3_^+^, a layered structure can be formed with inorganic germanium iodide layers separated by organic PEA layers (Figure 9c) [143]. The (PEA)_2_GeI_4_ HP revealed a direct bandgap of 2.12 eV making it suitable for tandem solar cells. This layered perovskite was also found to be much more stable in humid air than MAGeI_3_ because of its high hydrophobicity of the organic component [143].

The stability of MAGeI_3_ HP and its performance as a light harvester of solar cells can also be enhanced by introducing bromide additives. By substituting 10% I^−^ with Br^−^ a PCE of 0.57% was achieved (Table 1) in an inverted cell with a fullerene ETL [144].

The combination of Sn^2+^ and Ge^2+^ in single HPs results in solid-solution CH_3_NH_3_Sn_(1−_*_x_*_)_Ge*_x_*I_3_ compounds with a bandgap tunable from 1.3 eV (*x* = 0) to 2.0 eV (*x* = 1) [146].

A Mn^2+^-based analog of MAPI was produced by the spin-coating of a mixture of MnI_2_ and MAI on mesoporous titania scaffolds [147]. After covering with a Spiro-MeOTAD HTL, the MAMnI_3_-based device showed a response to the visible light illumination that was stable for at least 2000 s in an on/off cycling test [147]. A similar response to the UV light was observed for MA_2_MnCl_4_ perovskite incorporated into an FTO/TiO_2_/HP/carbon device [148].

One of the first Cu^2+^-based HPs (C_4_H_9_NH_3_)_2_CuCl_4_ was synthesized as early as in 2005 by reacting buthylamine hydrochloride with CuCl_2_ [149]. However, the potential of the hybrid perovskites was not yet realized at that time and this material was not tested as a potential light harvester.

A highly stable C_6_H_4_NH_2_CuBr_2_I compound was synthesized by reacting 2-iodaniline with CuBr_2_ [150]. It displayed extraordinary hydrophobicity and retained stability even after a 4 h immersion in water. This stability is coupled with a high sensitivity to visible light and a bandgap of 1.64 eV. A solar cell trial of this material showed a PCE of ≈0.5% [150] indicating plenty of room for further studies. A two-dimensional layered (C_6_H_5_CH_2_NH_3_)_2_CuBr_4_ perovskite (*E*_g_ = 1.81 eV) demonstrated a high stability and the feasibility for future photovoltaic applications [151].

**Figure 10:**
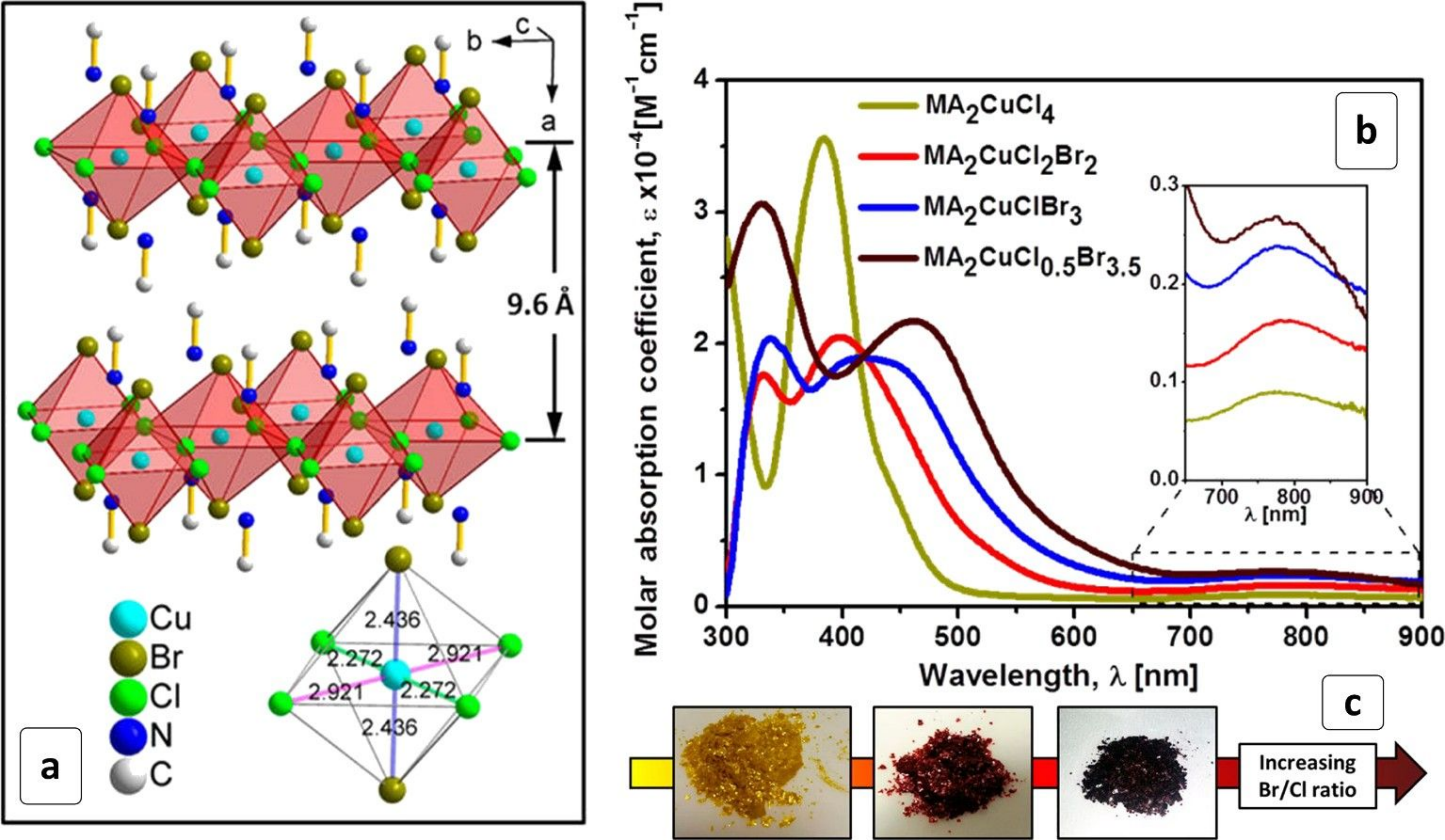
Structure (a), absorption spectra (b) and photographs (c) of (CH_3_NH_3_)_2_CuCl*_x_*Br_4−_*_x_* HPs of different composition. Reprinted and adapted with permission from [152], copyright 2016 American Chemical Society.

Recently, the first example of layered 2D copper-based (CH_3_NH_3_)_2_CuCl*_x_*Br_4−_*_x_* HPs has been reported [152] thereby demonstrating the appealing potential of such compounds for photovoltaic applications. The perovskite with *x* = 4 was studied in detail and found to be formed by single layers of CuCl_4_Br_2_ octahedra separated by cation-filled galleries with a size of ≈1 nm (Figure 10a). The materials are characterized by strong absorbance below 650 nm with absorption coefficients of ≈10^5^ cm^−1^ and a composition-dependent bandgap ranging from 2.48 eV for MA_2_CuCl_4_ to 1.80 eV for MA_2_CuCl_0.5_Br_3.5_ [152] (Figure 10b) and resulting in a gamut of HP colors from yellow to dark brown (Figure 10c).

#### Sb- and Bi-based hybrid perovskites

In contrast to Sn^2+^-based HPs that are prone to oxidation and hydrolysis, Bi- and Sb-based perovskites reveal a reasonable chemical/photochemical stability, retaining composition and structure in prolonged tests even without additional encapsulation. In the case of antimony, stable compounds of MA_3_Sb_2_I_9_ [90,153], MASbSI_2_ [87], and Cs_3_Sb_2_I_9_ [88–90,154] were reported, while for bismuth, a larger array of compositions was studied, including MA_3_Bi_2_I_9_ (MABI) [84–85,155–163], Cs_3_Bi_2_X_9_ [164–165], and MA_2_KBiCl_6_ [166]. The reported bandgaps of selected Bi- and Sb-based HPs are collected in Table 3.

**Table 3:** Bandgap and approximate absorption band edge position (λ_e_) of selected Bi- and Sb-based hybrid perovskites.

Perovskite	*E*_g_, eV	λ_e_, nm	Ref.

MA_3_Bi_2_I_9_	1.99 2.11 2.17 2.40	630 590 570 520	[155] [85] [86] [161]
(MA_2_)KBiCl_6_	3.04	410	[166]
MA_3_Sb_2_I_9_	1.80 1.95 2.14	690 640 580	[154] [90] [153]
MA_3_Sb_2_I_8_Cl	1.90	650	[154]
MA_3_Sb_2_I_7_Cl_2_	2.00	620	[154]
MASbSI_2_	2.03	610	[87]
Cs_3_Sb_2_I_9_	2.00 2.05	620 600	[90] [88]
Rb_3_Sb_2_I_9_	1.98	630	[91]

#### Bi-based HPs

The MABI perovskite is composed of Bi_2_I_9_ bi-octahedral units with two bismuth ions in the center of an octahedra connected via three iodine atoms. MABI shows two distinct electron transitions near the absorption band edge – an indirect transition with *E*_g_^i^ = 1.99 eV and a direct one with *E*_g_^d^ = 2.15 eV [155]. Both transitions contribute to the light-harvesting by the Bi-based HPs as indicated by detailed PL studies on luminescent Cs_3_Bi_2_X_9_ NCs [159]. A study of single-crystal and polycrystalline MABI showed that both materials have a long exciton lifetime and a high carrier mobility [161,163].

A transient absorption study of MABI crystals showed only a minor change of the exciton dynamics when the crystal size was reduced from micrometers to a few hundred nanometers [167]. A combination of MABI with a TiO_2_ scaffold resulted in a depopulation of bound excitons and electron transfer to the titania. These observations indicate that, in contrast to Pb-based HPs, for MABI, a bulk-heterojunction solar cell architecture is preferable to sub-micrometer HP domains.

Typically, the MABI-based solar cells demonstrate a high resistivity to air oxidation and ambient humidity [85,155–157,160,168]. Similar high stability to the degradation under ambient atmosphere was reported for highly luminescent Cs_3_Bi_2_X_9_ (X = Cl, Br, I) crystals emitting in a broad range from ≈390 to ≈550 nm depending on the composition of the halide component [164]. It is assumed that the moisture stability of the perovskites can originate from inherent self-passivation with a surface BiOX layer [164]. The water vapors were found to passivate the surface of Cs_3_Bi_2_X_9_ NCs resulting in a drastic PL enhancement [165]. The water was found to act similar to the addition of the surfactant oleic acid, confirming the assumption of the moisture-induced passivation of the surface trap states and providing the nanocrystalline Cs_3_Bi_2_X_9_ luminophores with prolonged stability [165]. Along with the chemical and photochemical stability, MABI retains perfect integrity during charging/discharging events. In particular, a MABI-based electrochemical capacitor retains around 85% of its initial maximal capacitance after more than ten thousand charge/discharge cycles [158].

A cell comprised on a MABI layer sandwiched between an FTO/TiO_2_ scaffold and a Spiro-MeOTAD/Au layer showed a PCE of 0.164% and very good stability of photovoltaic parameters even when stored in the open humid air [157]. The cell also showed almost no hysteresis over a broad range of scan rates (150–1500 mV/s). A hysteresis-free cell was also constructed by combining the single-crystalline MABI with a P3HT HTL [85].

**Figure 11:**
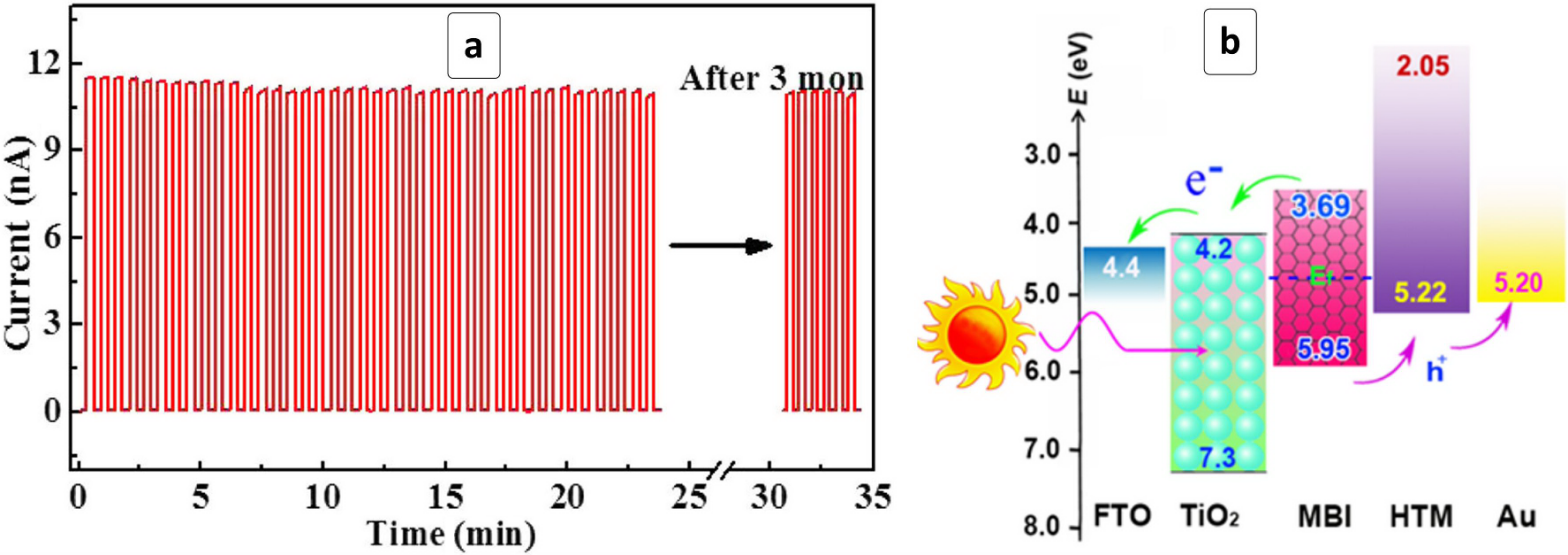
(a) Cyclic photoresponse of a red light photodetector based on CsBi_3_I_10_ HP with a freshly prepared photosensitive layer and after the three months of storage (last five cycles). Reprinted with permission from [169], copyright 2017 American Chemical Society. (b) Energy diagram of a solar cell based on MABI (MBI) HP, mesoporous TiO_2_ ETL and Spiro-OMeTAD HTL. Reprinted with permission from [84], copyright 2017 American Chemical Society.

A layer of CsBi_3_I_10_ perovskite deposited by a conventional spin-coating/drying method on top of gold electrodes demonstrated a high photoresponse in the range of λ < 700 nm [169]. The photodetector is characterized by an on/off ratio as high as ≈10^5^ and a prolonged stability retaining the unvaried response after a shelf-storage for at least three months (Figure 11a).

The efficiency of solar cells based on Cs_3_Bi_2_I_9_ HP nanosheets was found to depend on the composition of the HTL layer. The best PCE of 3.2% was observed for copper(I) iodide HTLs, a value claimed to be the highest achieved to date for the Bi-based HP family [170].

The morphology of MABI layers produced on titania scaffolds by a simple spin-coating of a BiI_3_+MAI mixture followed by a heat treatment was found to depend on the morphology of the scaffold, varying from island-like for the compact TiO_2_ layers to a more homogeneous MABI deposit on the mesoporous TiO_2_ [157].

The TiO_2_/MABI composites can be produced by a double-step interdiffusion method including sequential deposition of BiI_3_ and CH_3_NH_3_I layers followed by annealing at 100 °C [162]. The last step yields a much more uniform and homogeneous MABI layer than conventional single-step spin-coating/annealing resulting in almost doubled PCE.

Highly compact and pin-hole-free MABI films can be produced by a two-step process including the high-vacuum deposition of BiI_3_ followed by the conversion of bismuth triiodide into MABI [84]. The high quality of the HP layer resulted in a record PCE of 1.54% in a cell with a titania ETL and a Spiro-MeOTAD HTL (Table 1). The cell configuration allows for an efficient electron transfer from MABI to the TiO_2_ scaffold while the holes are withdrawn to the Spiro-MeOTAD HTL and then – into the gold back contact (Figure 11b). The charge separation efficiency is evidenced by a relatively high FF of almost 80%, while a high *V*_oc_ of 0.83 V observed for such cells attests to the structural perfection of the light-absorbing HP layer [84].

A similar *V*_oc_ (0.895 V) was reported for a MABI-based cell produced without HTLs with a single carbon back contact [86]. In this case, a top light conversion efficiency was only 0.054% (Table 1), indicating the crucial role of the hole transfer dynamics for the total cell performance.

The efficiency of MABI-based cells with the solution-processed HP layers is also limited by a rough interface between MABI and typical ETL/HTL materials. The interface quality can be increased by controlling the rate of MABI crystallization, in particular, by introducing additions of *N*-methylpyrrolidone (NMP) to DMF which is typically used as a solvent for the spin-coating deposition of MABI layers [160]. NMP slows the HP crystallization favoring the formation of a more uniform MABI layer and providing a ≈50% enhancement of the photocurrent generation efficiency. Simultaneously, the optimized morphology shows an enhanced stability, the cells retaining their characteristics after 30 days of exposure to ambient conditions (relative humidity of 50–60%) [160].

The structure and characteristics of (MA)_2_KBiCl_6_ perovskite [166] are very similar to MAPbCl_3_, however, the high bandgap of this material (3.04 eV) is more suitable for UV photodetectors than for the photovoltaic applications.

The organo–inorganic iodobismuthates C_5_H_6_NBiI_4_, C_6_H_8_NBiI_4_ and (C_6_H_13_N)_2_BiI_5_ displayed bandgaps of around 2 eV and stability under the ambient conditions [171–172]. Aromatic cations were found to contribute to the conduction band of these compounds, facilitating the transport of charge carriers. As a result, mesoscopic solar cells based on such iodobismuthates showed a PCE of ≈1% even without additional HTLs [171].

#### Sb-based hybrid perovskites

Antimony-based MA_3_Sb_2_I_9_ and Cs_3_Sb_2_I_9_ perovskites displayed bandgaps of 1.95 eV and 2 eV, respectively, and CB/VB positions suitable for most of ETL/HTL combinations (Figure 2d) [90]. Amorphous MA_3_Sb_2_I_9_ films were reported to have a bandgap of 2.14 eV and relatively high absorption coefficients of an order of 10^5^ cm^−1^ [153]. The films also demonstrated considerable sub-bandgap absorption with a characteristic Urbach energy of ≈60 meV, indicating a substantial level of structural and energetic disorder. Due to the disorder, planar inverted solar cells based on amorphous MA_3_Sb_2_I_9_ showed low photocurrent densities, however, with a relatively high open-circuit voltage (≈890 meV) and a decent fill factor (55%) [153], indicating the good potential of this light absorber for further studies and improvements. In particular, a careful control of the MA_3_Sb_2_I_9_ stoichiometry and introduction of HI additives during the film formation as well as an additional fullerene ETL into the solar cell configuration allowed for a PCE beyond 2% [90].

Cs_3_Sb_2_I_9_ HP has a bandgap of ≈2 eV and an intrinsic weak p-type conductivity [88,90]. The energies of lowest direct (*E*_g_^d^) and indirect (*E*_g_^i^) electron transitions differ only by ≈0.02 eV and both transitions are characterized by absorption coefficients similar to those of MAPI [88].

Cs_3_Sb_2_I_9_ can exist as two polymorphs – a layered (2D) modification built by two-dimensional layers of polyanions and a “dimer” (0D) form built of isolated dioctahedral Sb_2_I_9_^3−^ anions [89]. The HP formation can be directed to one of these forms by tailoring the temperature of annealing, the 0D and 2D modifications forming at 150 and 250 °C, respectively. The layered 2D form demonstrated higher electron and hole mobilities and a better tolerance to defects as compared to the dimer-built 0D polymorph. The 2D-Cs_3_Sb_2_I_9_ HP can be formed by annealing the original metal iodide mixture at 250 °C provided that a portion of SbI_3_ lost during the heating due to a high SbI_3_ vapor pressure at the annealing temperature is compensated [89]. A comparative study of inverted solar cells composed of 2D- and 0D-Cs_3_Sb_2_I_9_ (produced at 150 °C), a PEDOT:PSS HTL and a PC_70_BM ETL resulted in PCEs of 1.5% and 0.89% for 2D- and 0D-form, respectively [89]. This finding, along with a higher stability of 2D-HP-based devices, suggests that the layered form of Cs_3_Sb_2_I_9_ is preferable for photovoltaic applications.

**Figure 12:**
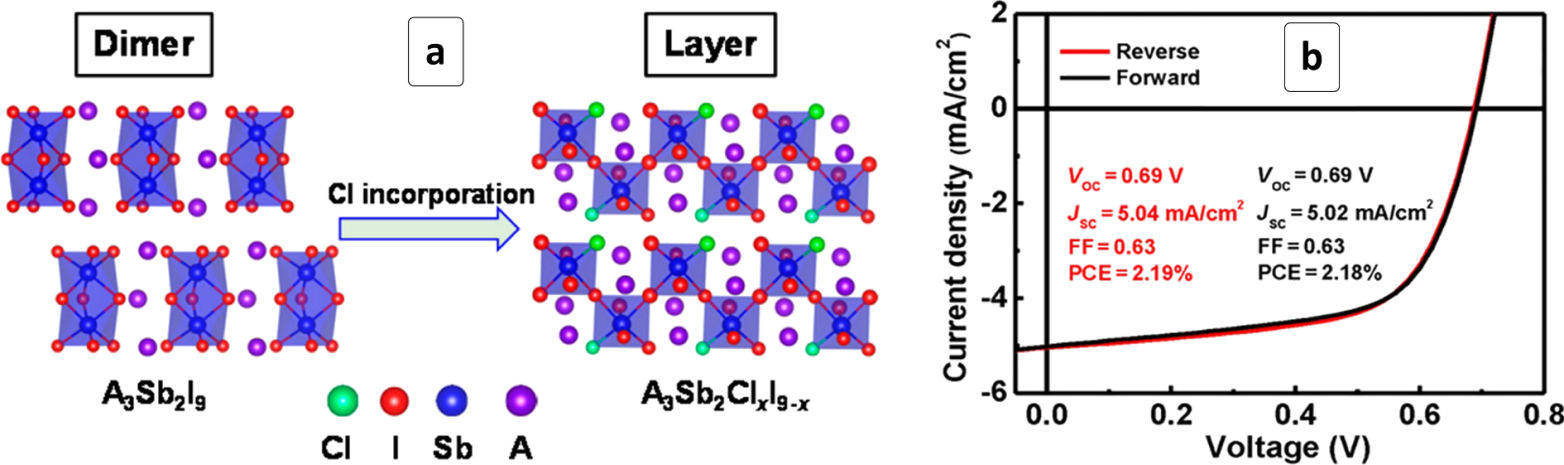
Schematic structure of MASb_2_Cl*_x_*I_9−_*_x_* HP (a) and current–voltage characteristics of a solar cell based on MA_3_Sb_2_Cl*_x_*I_9−_*_x_* HP registered at a forward and reverse bias (b). Reprinted and adapted with permission from [154], copyright 2018 American Chemical Society.

The incorporation of other halide ions allows the crystal structure and optoelectronic properties of MA_3_Sb_2_I_9_ HP to be altered. In particular, the introduction of chloride results in a transformation of dimer-like MA_3_Sb_2_I_9_ built of bi-octahedral Sb_2_I_9_ units into a layered MA_3_Sb_2_Cl*_x_*I_9−_*_x_* compound where antimony iodide octahedra are bound to infinite chains via a shared I^−^ anion (Figure 12a) [154]. Simultaneously, the bandgap broadens from 1.8 eV (MA_3_Sb_2_I_9_) to 2.0 eV for MA_3_Sb_2_Cl_2_I_7_. Solar cells comprising such 2D MA_3_Sb_2_Cl*_x_*I_9−_*_x_* HPs displayed PCEs surpassing 2% (Table 1), while the highest PCE reported for “conventional” Sb-based halide HPs is lower than 1% [154]. Similar to the Bi-based HPs, such cells demonstrated good stability in the humid air environment and no hysteresis between *C*–*V* curves obtained at a direct and reverse potential scans (Figure 12b).

**Figure 13:**
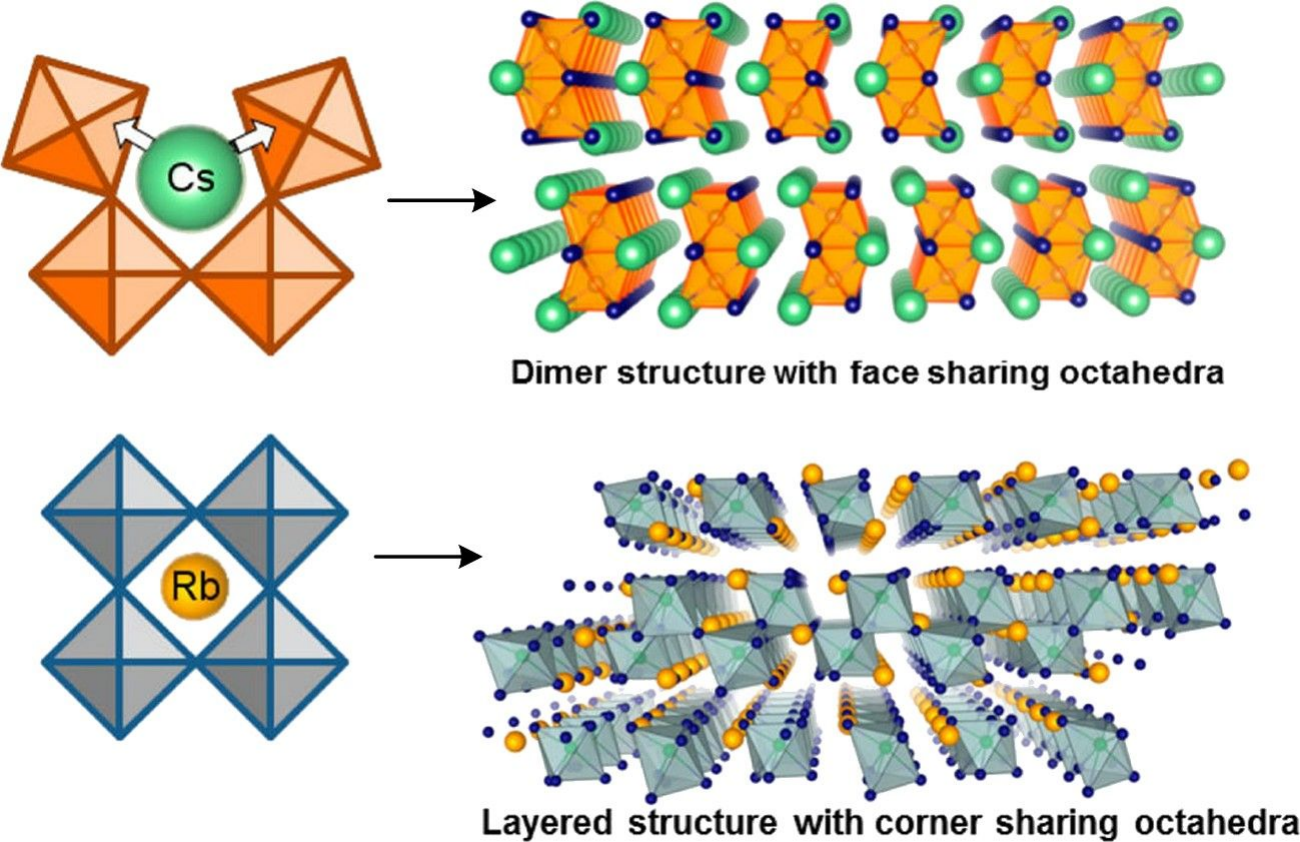
Schematic of the influence of the cation size on the structure of A_3_Sb_2_I_9_ (A = Cs, Rb). Reprinted and adapted with permission from [91], copyright 2016 American Chemical Society.

The Sb_2_I_9_ dimer-based modification of Sb-HPs is typical for the compounds with both bulky organic cations and Cs^+^ and forms as a result of steric factors (Figure 13a). As this modification has a relatively low charge transport efficiency, various approaches are developed to forward the HP formation to other modifications, including the above-discussed introduction of Cl^−^ ions as well as the replacement of bulky cations with smaller ones, for example, with Rb^+^ [91]. The rubidium cations can be accommodated by the layered modification (Figure 13b) and the latter forms irrespective of the HP synthesis method [91]. The Rb_3_Sb_2_I_9_ HP is characterized by a bandgap of 1.98 eV and a higher (less negative) CB position as compared to the MA^+^ and Cs^+^-based counterparts, which is favorable for the electron transfer to typical ETL materials (Figure 2d).

**Figure 14:**
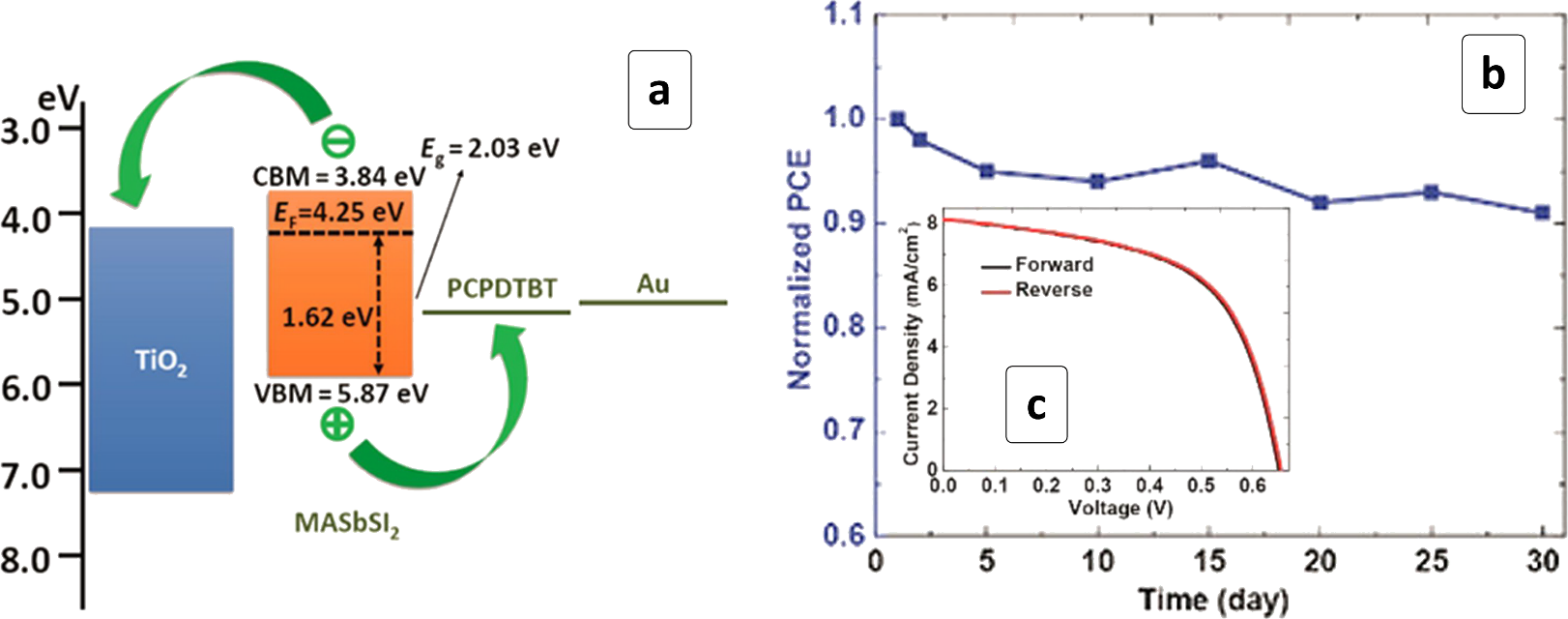
Energy diagram (a), normalized IPCE tested for a period of 30 days (b) as well as current–voltage characteristics registered at forward and reverse bias (c) of a solar cell based on MASbSI_2_ HP. The PCPSTBT abbreviation is explained in the notes of Table 1. Reprinted and adapted with permission from [87], copyright 2018 American Chemical Society.

Using a combination of halide and chalcogenide anions for building an organo–inorganic pHP structure potentially allows for the introduction of central metal ions in the oxidation states of +3 and +4. This idea was realized in the case of MASbSI_2_ HP produced by a sequential stepwise method by reacting Sb_2_S_3_ with SbI_3_ and then with MAI on the surface of a mesoporous TiO_2_ scaffold [87]. The MASbSI_2_ HP displayed a bandgap of 1.62 eV and CB/VB positions suitable for the electron/hole transport to TiO_2_ and a variety of HTL materials, respectively (Figure 14a). A MASbSI_2_-based solar cell demonstrated a PCE of more than 3% (Table 1), in addition to a reasonable stability in a 30-day trial (Figure 14b) and no discernible hysteresis between the forward and reverse *C*–*V* scans (Figure 14c) [87].

Recently it was found that organo–inorganic bromoantimonate (*N*-ethylpyridinium)_3_SbBr_6_ forms perovskite-like 3D crystalline framework compounds that have great potential as solar cell absorbers [173]. A solar cell based on the particular (*N*-ethylpyridinium)_3_SbBr_6_ displayed an external quantum efficiency of ≈80% and a PCE of ≈4% thus coming into the same league as lead-halide HPs [173].

A series of (*N*-methylpyrrolidinium)_3_Sb_2_Cl_9−9_*_x_*Br_9_*_x_* (*x* = 0−1) HPs was reported to have bandgaps tunable from 2.76 eV (*x* = 1) to 3.31 eV (*x* = 0) and to exhibit photosensitivity levels high enough for the application of such compounds in UV–vis photodetectors [174].

The search for new lead-free HPs based on M^3+^ cations continues on. It often starts from the theoretical estimation of the stability, structure and optoelectronic properties of various HPs and selection of the most promising ones, stimulating further experimental work. For example, a broad theoretical screening of various mono- and bi-metallic lead-free perovskites among more than 480 materials focused on the 10 most promising in terms of the bandgap. Among these, the smallest *E*_g_ was found for Cs_3_Ga_2_I_9_ (*E*_g_ = 1.72 eV) [175], which is still to be synthesized and tested in photovoltaic applications.

A series of HPs based on lanthanide cations was modeled and Eu-, Dy-, Tm-, and Yb-based HPs selected as the materials with the most promising bandgaps in the range of 2.0–3.2 eV [176]. It was found that localized f-electrons of lanthanide ions have a strong contribution to the VB top, where a strong influence of the lanthanide ion on the properties of HPs is expected. A combination of a theoretical screening with a following experimental verification recently led to a series of Cs_2_TiI*_x_*Br_6−_*_x_* HPs with bandgaps varying from 1.38 eV to 1.78 eV depending on the I/Br ratio [177].

#### Lead-free hybrid perovskites based on A^+^/A^3+^ metal compositions

**Ag-Bi, Ag-Sb:** A series of stable lead-free bimetallic HPs with a cation pair aliovalent to a pair of Pb^2+^ (Sn^2+^) ions such as A_2_AgBiX_6_, A_2_AgSbX_6_, and A_2_AgInX_6_ (A = MA or Cs, X = Cl, Br) was reported. The bandgaps of selected A^+^/A^3+^-HPs are presented in Table 4.

**Table 4:** Bandgap and approximate absorption band edge position (λ_e_) of selected A^+^/A^3+^-based HPs.

Perovskite	*E*_g_, eV	λ_e_, nm	Ref.

MA_2_AgInI_6_	1.93	640	[178]
Cs_2_AgInCl_6_	3.2–3.3	380–390	[179]
Cs_2_AgBiCl_6_	2.77 2.20	450 560	[180] [181–182]
Cs_2_AgBiBr_6_	1.90–1.95 2.19–2.25	640–650 550–570	[182–184] [150,185–186]
MA_2_AgBiBr_6_	2.02	610	[187]
MA_2_AgBiI_6_	1.96	630	[188]
Cs_2_Au_2_I_6_	1.31	950	[189]

**Figure 15:**
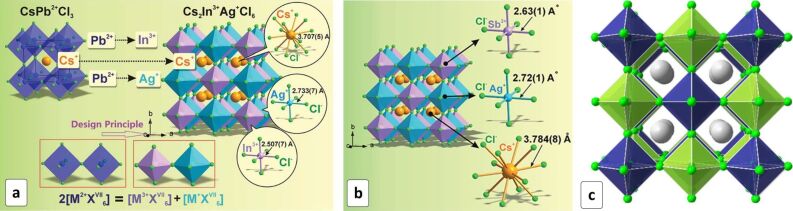
(a) Evolution of the crystal structure from CsPbCl_3_ to Cs_2_AgInCl_6_ HP; (b) Crystal structure of Cs_2_AgSbCl_6_ HP; (c) Crystal structure of Cs_2_AgBiCl_6_ HP; Cs^+^ ions are presented as gray spheres and Cl^–^ as green spheres, the Ag and Bi centered octahedra are shown as blue and green ones, respectively. (a) Reproduced with permission from [179], copyright 2017 The Royal Society of Chemistry; (b) Reproduced with permission from [190], copyright 2018 The Royal Society of Chemistry; (c) Reprinted with permissions from [180], copyright 2016 American Chemical Society.

Cs_2_AgInCl_6_ HP was produced by a hydrothermal synthesis allowing the size of the final crystallites to be tailored by adjusting the hydrothermal treatment duration [179]. The perovskite crystallizes in the rock-salt structure with alternating [AgCl_6_] and [InCl_6_] octahedra (Figure 15a) and shows a direct bandgap of 3.23 eV, as well as an excellent stability to the ambient moisture, light, and heat [179].

Cs_2_AgSbCl_6_ HP (Figure 15b) [190] and Cs_2_AgBiCl_6_ HP (Figure 15c) [180–181] are isostructural to Cs_2_AgInCl_6_ and display comparable photochemical, hydrolytic and thermal stability.

Despite a more complex composition as compared with the Pb-based analog, the Cs_2_AgInCl_6_ HP can be prepared with a trap state density of ≈10^9^ cm^−3^, which is much lower than that reported for the best lead halide HPs [191]. Such close-to-ideal materials can be used as light-absorbing bodies of very sensitive, stable and fast UV photodetectors. The doping of Cs_2_AgInCl_6_ HPs with Mn^2+^ imparts this HP with the property of visible-range luminescence [192].

Theoretical studies predicted Cs_2_AgInBr_6_ HP to be thermodynamically stable [193–194], showing a distinct n-type conductivity and shallow trap levels for Ag-rich and Br-poor compositions [194].

MA_2_AgSbI_9_ HP was found to be a stable compound both by DFT calculations and experimental evidence, showing excellent resistivity to the ambient air/humidity [178]. The material revealed a band gap of 1.93 eV and CB/VB positions (−6.28 eV/−4.35 eV) suitable for most ETL/HTL materials used in the HP solar cells.

The substitution of chloride ions with Br^−^ in Ag-Bi-based HP results in a shrinking of the bandgap from 2.77 eV to 2.19 eV [180], both values being smaller than *E*_g_ of corresponding Pb-based HPs. The electron transitions contributing to the absorption band edge were found to be of an indirect character in accordance with DFT predictions [182,195–196] with the calculated bandgaps of 2.2 eV [181–182] and 1.9 eV [182] for the Cl-based and Br-based HPs, respectively. The calculations also showed that a partial substitution of silver(I) with copper(I) should result in a narrowing of the bandgap to 1.6–1.9 eV, which would not compromise the HP stability [197], but these conclusions are still to be verified experimentally.

A DFT modeling of TiO_2_/Cs_2_AgBiX_6_ (X = Cl, Br) interfaces showed them to be very favorable for the photoinduced charge separation due to an appropriate band alignment and a state density gradient along the interface [198]. The calculations also showed that the bandgap of Cs_2_AgBiBr_6_ HP can be continuously tuned from 1.93 eV down to 0.44 eV by introducing a control lattice disorder the latter value characteristic of completely disordered alloy [183]. As a practical means to exert such a disordering, the controlled doping of the perovskite was proposed [183]. However, such disordering will inevitably limit the charge transport efficiency and boost the electron–hole recombination and, therefore, a certain equilibrium between the light harvesting capability and the photovoltaic efficiency can be expected in such approach.

The bandgap of Cs_2_AgBiBr_6_ HP was also found to decrease upon application of a high-pressure treatment [185]. Under 15 GPa pressure, the AgBi-based perovskite displays a bandgap of ≈1.7 eV (≈22% lower than at the ambient pressure) close to the *E*_g_ of the “classical” MAPI, retaining about 8% residual *E*_g_ shrinkage after the pressure is released [185].

The results of the DFT simulations of the electron structure of more than 11,000 various compositions of lead-free HPs were assembled as a material database available for the screening of possible light harvesters of the photovoltaic solar cells [199]. A comprehensive theoretical screening of more than 480 A_2_MM'X_6_, AMX_4_, and A_3_M_2_X_9_ compounds put the focus on the ten most promising materials with bandgaps between 1.5–2.5 eV [175].

Time-resolved microwave conductance studies of Cs_2_AgBiBr_6_ HP in the form of thin films and crystals revealed the presence of mobile charges with lifetimes on the order of microseconds as well as a low level of trap-state-mediated recombination, which is promising for the photovoltaic applications of this material [200].

Similar to MABI, Cs_2_AgBiBr_6_ perovskite showed a remarkable tolerance to a variation of the grain size and the defect density, maintaining an unchanged PL yield both for the bulk crystal and finely powdered samples [183]. These findings show the feasibility of the design of bulk heterojunction solar cell architectures with nanometer- and micrometer-sized HP domains.

The performance of Cs_2_AgBiBr_6_ HP-based solar cells is typically limited by the low quality of the HP layer, resulting from the poor solubility of the Ag and Bi halide precursors. To address this problem, a variety of solvents and deposition modes were tested and an approach to produce very uniform Cs_2_AgBiBr_6_ films was proposed by exploiting DMSO as a “universal” solvent and a special series of thermal treatments of both the precursor solutions and spin-coated films [186]. The solar cells with the optimized films sandwiched between a TiO_2_ ETL and a Spiro-MeOTAD HTL showed a PCE of 2.43% and an open-circuit voltage of almost one volt, which is the highest value currently reported for Bi-based HPs. The devices also displayed a remarkable stability in working conditions even without additional encapsulation [186].

The superior stability of Cs_2_AgBiBr_6_ HP as compared to MAPI was rationalized by a detailed theoretical structural analysis [201] that showed the framework of the AgBi-HP to be much more rigid with considerably lower thermal expansion coefficients as a result of the presence of relatively stronger Ag–Br and Bi–Br bonds.

**Figure 16:**
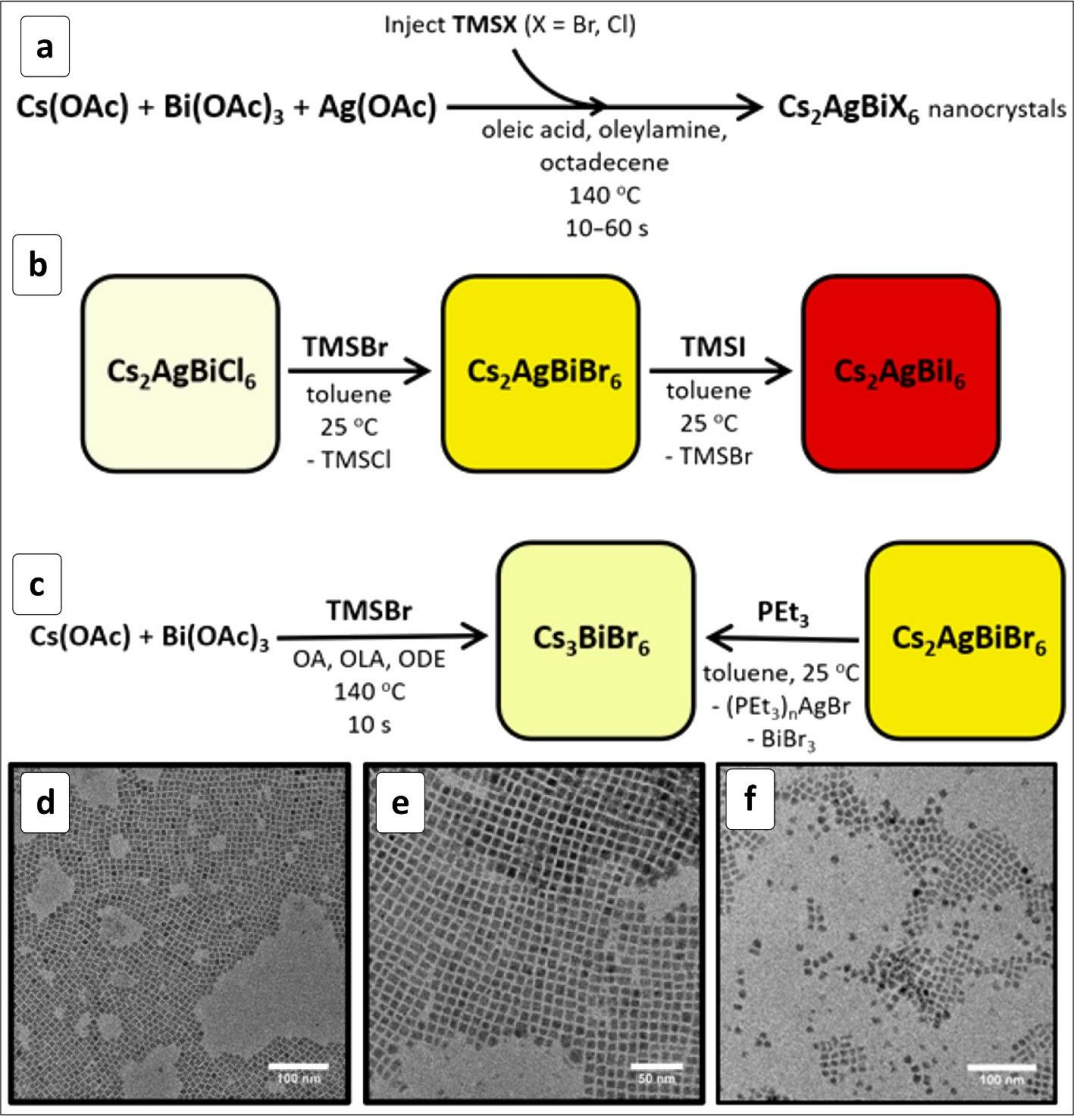
Scheme of the synthesis of Cs_2_AgBiX_6_ NCs (a–c) and TEM images of Cs_2_AgBiCl_6_ (d), Cs_2_AgBiBr_6_ (e), and Cs_2_AgBiI_6_ NCs (f). OAc is acetate anion, Et – ethyl, OA – oleic acid, OLA – oleylamine, ODE – octadecene, TMS – trimethylsilyl. Reprinted and adapted with permission from [202], copyright 2018 American Chemical Society.

The Cs_2_AgBiCl_6_ and Cs_2_AgBiBr_6_ NCs synthesized by a hot-injection approach (Figure 16a) can be converted to the Cs_2_AgBiI_6_ phase either by exchanging Cl(Br) with iodide anions (Figure 16b) or by exchanging Cs^+^ cations in Cs_3_BiBr_6_ NCs with Ag^+^ (Figure 16c) [202]. The NCs preserve the average size (≈8 nm) and size distribution (Figure 16d–e) during the ion-exchange conversions, showing a good morphology control provided by this method. The indirect band gap of Cs_2_AgBiX_6_ NCs decreases from ≈2.8 eV for X = Cl to ≈2.2 eV for X = Br to ≈1.75 eV for X = I [202].

Stable MA_2_AgBiBr_6_ HP was produced by a hydrothermal treatment of a mixture of MABr, BiBr_3_, AgBr, and HBr in water [187]. The formation of the bimetallic HP proceeds, most probably, via a step of the MA_3_Bi_2_Br_9_ phase formation, which is present as a residual in the final MA_2_AgBiBr_6_ HP, making this approach similar to the above-discussed ion exchange method. Additionally, the authors note the unsuccessful attempts to apply the hydrothermal method for the synthesis of (MA)_2_AgBiI_6_ and (FA)_2_AgBiBr_6_, the reaction yielding only the monometallic Bi-based phases [187]. The MA_2_AgBiBr_6_ HP revealed an indirect bandgap of ≈2 eV close to the range reported for the Cs-based analog (1.95–2.19 eV) [187].

The lead-free MA_2_AgBiI_6_ HP was synthesized by a solid-state reaction and revealed an indirect bandgap of 1.96 eV and a high stability to air exposure [188].

A broad theoretical modeling of the structures and properties of A_2_M^+^M^3+^X_6_ HP materials focused on two prospective materials with a direct bandgap of around 1 eV – Cs_2_InSbX_6_ and Cs_2_InBiX_6_ [203]. However, experimental attempts to exchange Ag^+^ to In^+^ in Cs_2_AgBiBr_6_ HP were unsuccessful due to the ready oxidation of In^+^ to In^3+^ [204]. At the same time, theoretical calculations [204] predicted that introduction of bulky MA or FA cations will stabilize In^+^, providing the compound with the optoelectronic properties close to those of MAPI and, therefore, a further pursuit in this direction may be fruitful. DFT calculations also indicated that MATl_0.5_Bi_0.5_I_3_ may be a potential candidate for a solar cell absorber with properties similar to those of MAPI [205].

Recently, a new Cs_2_Au_2_I_6_ HP was reported to have crystallized in a distorted tetragonal mix-valence Au^+^/Au^3+^ perovskite structure with a “close-to-ideal” bandgap of 1.31 eV [189]. Preliminary tests showed high promise of this material for thin-film photovoltaics.

### Conclusion and outlook

The present review shows that lead-free hybrid perovskites combine the many possibilities in composition with environmentally benign constituents, in addition to being relatively robust against the influence of light, air and moisture, and can be synthesized in a variety of possible morphologies (0D, 1D, 2D, etc.). The progress in this area is evident with the highest PCEs achieved to date already comparable and competitive with the efficiency of “conventional” lead-based perovskites. This progress is even more pronounced on the background of a saturation of the studies of Pb HPs and the light conversion efficiency achieved. The lead-free HP-based solar cells could soon become competitive to their Pb-based analogs in the near future because even with an inferior PCE, lead-free HPs are not acutely toxic and do not pose the severe concerns of possible environmental damage and the post-utilization recycling problems that Pb-based HPs do.

To increase the competitiveness of lead-free HP-based solar cells, several vital issues should be addressed. Among them – an inferior efficiency of charge transport, high recombination losses, and a limited spectral sensitivity range that is typical for such materials. The most promising avenues for addressing the charge separation/transport issues seem to be in an adapted morphological design of lead-free HPs, in particular, by implementing nanodimensional and layered architectures. The limitations in spectral sensitivity can be solved by designing composite light-harvesting systems with other semiconductors, such as narrow-bandgap Cd- and Pb-free binary and multinary metal chalcogenides.

In contrast to Pb-based HPs, the lead-free analogs typically display a relatively short-range free charge migration distance on the order of several tens of nanometers. In this view, a decrease of the HP crystal size to 10–100 nm should not fundamentally affect the charge transport properties but might open broad possibilities of increasing the efficiency of interfacial electron transfer and allow the construction of “ideal” bulk heterojunctions with ETL and HTL materials by intermixing nanometer-sized particles of components. In this view, the lead-free HPs can potentially be utilized in the nano-dispersed form, differing drastically from Pb-HPs where a larger HP grain size and a smaller grain boundary typically mean a higher light conversion efficiency.

The utilization of nanometer building blocks for the design of lead-free HP-based solar cells requires reliable synthetic methods allowing for a precise control over the HP morphology. In this area, conventional synthetic strategies are typically used, and the HP NCs are grown in high-boiling-point solvents in the presence of surface-passivating ligands. These approaches seem to be quite appropriate for the synthesis of highly luminescent perovskites. However, for photovoltaic applications, new methods are needed to produce NCs with a free surface or containing only small passivating ligands as well as to form various composites; in particular, HP NCs supported on mesoporous metal oxide scaffolds. In this view, of particular significance are methods based on the formation of metal NCs as precursors for development of perovskite NCs. The metal NCs can be deposited by a variety of methods, but only the evaporative deposition of Pb and Sn NCs followed by their transformation into corresponding HPs has been probed (recently by Hodes et al. [136]). The transformation of metal NCs into perovskites was found to yield light absorbers of a higher quality and allowed for better morphology control as compared to the conventional spin/cast dropping deposition. At the same time, this method excludes toxic (Pb) or unstable (Sn) precursors. This strategy may be considered as very promising because metal NCs can be deposited by a variety of methods (electrochemical/electrocatalytic deposition, photochemical/photocatalytic deposition, thermal evaporation, “seed” growth on pre-deposited nuclei, etc.). This could allow for a precise method of control over the metal NC size that is suitable both for single metals (Sn, Sb, Bi, etc.) and for bimetallic alloys (Ag–Bi, Ag–Sb, Ag–In, etc.) that can then be converted into a plethora of hybrid perovskites. The feasibility of the exact translation of the morphology of primary metal NCs into the morphology of the final HPs still remains to be explored.

Layered 1D and 2D HP materials show great promise due to a strong anisotropy of electron properties, facilitating the charge separation and transport, as well as the unique morphological variability [57–58,64]. The latter can be achieved both by introducing various interlayer additives and by varying the thickness and composition of the layers themselves. For example, a strong effect on the light absorption, electron mobility, and internal electric field was predicted for ultra-thin CsSnI_3_ HP with the layer number as small as 1–3 [206]. The thickness dependence seems to resemble that of layered metal dichalcogenides, MoS_2_ and WS_2_, where single and few-layer materials differ drastically from corresponding bulk semiconductors. We may expect similar effects arising for layered lead-free HPs, potentially contributing to new designs of solar cells and enhanced light conversion efficiencies [14,207].

Simultaneously, the formation of interlayer voids or intermediate layers of other semiconductors/dielectrics may result in a regular quantum-well structure that is strongly beneficial for the photoinduced charge separation between the separated HP sheets [207–208]. Finally, a combination of single layers of two and more different lead-free HPs into a composite material may offer unprecedented variability of optical properties and band design. The feasibility of such an approach was probed by DFT calculations for Cs_3+_*_n_*M*_n_*Sb_2_X_9+3_*_n_* (M = Sn, Ge) compounds formed by inserting variable [SnI_6_] or [GeI_6_] octahedral layers into the [Sb_2_I_9_] bilayers. It was found, in particular, that adjusting the thickness of the inserted octahedral layers is an effective approach to tune the bandgap and effective mass of the charge carriers over a broad range [209].

Except for Sn-based HPs, the lead-free perovskites typically have larger bandgaps than “conventional” Pb-based compounds and, therefore, a limited spectral sensitivity range. This limitation can be avoided by designing tandem solar cells, for example with silicon or kesterite Cu_2_ZnSn(S,Se)_4_ absorbers. The feasibility of such an approach was demonstrated in 2014 by Todorov et al. who reported a relatively simple tandem solar cell based on a planar heterojunction between microcrystalline layers of kesterite and MAPI that displayed an unprecedented high *V*_oc_ of 1350 mV and a PCE exceeding 20% [210].

Alternatively, the lead-free HPs can be directly coupled with broadband-absorbing inorganic NCs to give a double benefit of extended spectral sensitivity range and a possibility of the photoinduced electron/hole separation between HP and inorganic NCs. Such systems have recently emerged as a new research hotspot, the attention focused mainly on combinations of PbS or PbSe NCs with Pb-based perovskites. In particular, PbS NCs introduced into MAPI-based solar cells were found to act simultaneously as a co-absorber and an HTL, rendering the use of additional organic hole transporting materials superfluous [211–212]. A shell of MAPI on the surface of PbS NCs was found to act as an efficient electron acceptor and a passivating agent, minimizing the recombinational losses in lead sulfide NCs [213–214].

To date, no reports can be found on analogous systems comprising lead-free HPs and cadmium- and lead-free semiconductors, such as CuInS(Se)_2_ or Cu_2_ZnSnS(Se)_4_, where both an “ideal” spectral sensitivity range and an efficient charge carrier separation between n-conducting HPs and p-conducting metal chalcogenides can be simultaneously achieved. This venue seems to be especially promising in view of recent developments in the synthetic approaches to both multinary metal chalcogenide NCs and lead-free HP NCs with highly controlled composition and morphology. We may expect that the combination of nanometer building blocks of inorganic semiconductors and hybrid perovskites will allow for the construction of “green” and efficient bulk-heterojunction configurations with a panchromatic spectral response and competitive light conversion efficiencies.
